# Integrated Foodomics Reveals Gut Microbiota–Metabolite–Gene Interactions Associated with the Immunoprotective Effects of Ganoderma lucidum Polysaccharide Peptide

**DOI:** 10.3390/foods15132370

**Published:** 2026-07-03

**Authors:** Jing Xie, Zilong An, Dongmei Lin, Jing Li, Shuqi Yu, Mazurenko Ihor, Zhanxi Lin

**Affiliations:** 1 College of Agriculture and Biotechnology, Hunan University of Humanities, Science and Technology, Loudi 417000, China0487222489@ukr.net (M.I.); 2 Health Food Technology Research and Verification Center, Hunan University of Humanities, Science and Technology, Loudi 417000, China; 3 National Engineering Research Center of Juncao Technology, Fujian Agriculture and Forestry University, Fuzhou 350002, China

**Keywords:** *Ganoderma lucidum* polysaccharide peptide, cyclophosphamide-induced immunosuppression, immune regulation, gut microbiota, foodomics, functional food ingredient

## Abstract

*Ganoderma lucidum* polysaccharide peptide (GLPP) is a food-derived macromolecule with immunomodulatory potential, but its gut-centered mechanisms under chemotherapy-associated immunosuppressive stress remain unclear. This study aimed to evaluate the protective effects of GLPP against cyclophosphamide (CTX)-induced immunosuppression and intestinal injury in mice and to explore the associated microbiota–metabolite–gene interaction network using integrated foodomics. BALB/c mice were treated with CTX and then administered GLPP at 50, 100, or 200 mg/kg/day for 42 days, with levamisole as a positive control. High-dose GLPP restored spleen index from 1.592 ± 0.266 to 1.902 ± 0.212 mg/g and thymus index from 0.322 ± 0.146 to 0.656 ± 0.081 mg/g compared with the CTX group. It also enhanced lymphocyte proliferation (OD450: 1.529 ± 0.073 vs. 1.065 ± 0.051), increased carbon clearance index (3.403 ± 0.223 vs. 2.650 ± 0.164), elevated IL-2 and IgA levels, and reduced excessive IFN-γ and TNF-α responses. GLPP alleviated intestinal mucosal injury and reshaped gut microbial profiles, particularly taxa related to Bacteroidota and *Bacteroides*. Metabolomics revealed putatively annotated differential metabolites associated with amino acid, nicotinate–nicotinamide, and glycerophospholipid metabolism, while transcriptomics indicated modulation of PRR/MAPK-related immune signaling. Integrated correlation analysis suggested a microbiota–metabolite–gene–immune association network involving putative gamma-Glutamylleucine(γ-Glu-Leu), leukotriene D4(LTD4)-like lipid features, and hippuric acid. These findings support GLPP as a promising immune-supporting functional food ingredient, although metabolite assignments and causal mechanisms require further validation.

## 1. Introduction

*Ganoderma lucidum* (*G. lucidum*) is a well-known edible and medicinal basidiomycete with a long history of dietary and medicinal use [1]. Its biological activities have mainly been attributed to triterpenes, polysaccharides, and polysaccharide–peptide conjugates [2,3]. As a characteristic macromolecular component of *G. lucidum*, *Ganoderma lucidum* polysaccharide peptide (GLPP) contains approximately 87.17% polysaccharides and 5.04% peptides, includes 16 amino acids, and has a molecular weight of approximately 5 × 10^4^ Da [4]. Previous studies have shown that GLPP exerts anti-atherosclerotic, antioxidant, anti-inflammatory, and immunomodulatory activities, including the regulation of macrophage polarization and immune-related cytokines [5,6]. Because *G. lucidum* is widely used as a functional food and nutraceutical raw material, GLPP has potential as a food-derived ingredient for immune support. Previous toxicological studies have indicated that *G. lucidum* polysaccharides and related glycopeptides, including GLPP, exhibit low toxicity and a favorable safety profile in animal models [7,8,9]. However, its development is still limited by incomplete understanding of how its carbohydrate–peptide structure interacts with the intestinal ecosystem, microbial metabolism, and host immune regulation [10,11,12]. Therefore, the central biological question of this study is how does GLPP, as a food-derived polysaccharide–peptide conjugate, protect against chemotherapy-associated immunosuppression and intestinal injury through a gut-centered mechanism? Clarifying these interactions from a foodomics perspective is therefore important for linking GLPP composition with its potential health-promoting function.

We hypothesized that GLPP restores immune homeostasis by reshaping the gut microbiota, which in turn modulates immunologically active metabolites and host gene expression pathways. Foodomics provides an integrated framework for understanding how food-derived bioactive components influence host health through coordinated changes in gut microbiota, metabolites, and gene expression. In this study, integrated foodomics refers to the combined use of 16S rRNA gene sequencing, ultra-performance liquid chromatography–tandem mass spectrometry (UPLC-QTrap-MS/MS)-based metabolomics, and cecal transcriptomics to investigate GLPP-mediated food–host–microbe interactions. This approach is especially suitable for GLPP, because polysaccharide–peptide conjugates may exert broad biological effects through both microbial fermentation and host immune signaling. Unlike single-target pharmacological agents, dietary macromolecules often regulate health through multi-level intestinal and systemic pathways.

Single-omics approaches have advanced mechanistic research but provide only partial information about the biological effects of food-derived bioactive components [13,14]. Metabolomics captures metabolic changes but does not identify upstream microbial or gene-regulatory drivers [15,16]; transcriptomics reveals gene expression responses but does not directly reflect metabolite-mediated physiological changes [17]; and microbiomics describes host–microbe interactions but cannot alone connect microbial shifts to host molecular pathways [18,19]. The integration of microbiomics, metabolomics, and transcriptomics can therefore provide a more comprehensive strategy for identifying key regulatory nodes linking gut microbiota, host metabolism, gene expression, and immune phenotypes [20,21,22,23,24,25,26,27,28,29,30,31,32]. Although multi-omics strategies have been applied to edible fungus polysaccharides and food-derived peptides [33,34,35,36,37,38], to our knowledge, no study has systematically integrated gut microbiome, metabolome, and cecal transcriptome data to address the specific question of GLPP-mediated microbiota–metabolite–gene–immune crosstalk under CTX-induced immunosuppressive stress.

Therefore, we established a CTX-induced immunosuppressed mouse model to evaluate the immunoprotective effects of GLPP and to explore its underlying gut-centered regulatory mechanisms. The objectives were to (1) evaluate the effects of GLPP on immune organ indices, immune cell-related functional assays, cytokine/IgA levels, and intestinal morphology; (2) identify GLPP-associated changes in gut microbiota, cecal metabolites, and cecal gene expression; and (3) integrate microbiome, metabolome, and transcriptome data to construct a microbiota–metabolite–gene–immune network associated with GLPP-mediated immune restoration. The study provides mechanistic evidence supporting GLPP as an immune-supporting functional food ingredient.

## 2. Materials and Methods

### 2.1. Chemicals and Reagents

GLPP powder (molecular weight ≈ 50 kDa, purity ≥ 95%) was provided by the National Engineering Research Center of Juncao Technology (Fuzhou, China). The GLPP used in this study contained approximately 87.17% polysaccharides and 5.04% peptides, as reported previously [4]. Its monosaccharide composition was analyzed by gas chromatography–mass spectrometry (GC-MS), and its peptide sequence was identified by liquid chromatography–mass spectrometry (LC-MS/MS), confirming the presence of 16 amino acids [4]. Cyclophosphamide (CTX, Cat. No. C849559) was purchased from Shanghai Macklin Biochemical Co., Ltd. (Shanghai, China). Levamisole hydrochloride (LMS, Cat. No. L8230), used as the positive control drug, was obtained from Beijing Solarbio Science & Technology Co., Ltd. (Beijing, China). Enzyme-linked immunosorbent assay (ELISA) kits for immunoglobulin A (IgA), interleukin-2 (IL-2), tumor necrosis factor-α(TNF-α), and interferon-γ(IFN-γ) were purchased from Wuhan ABclonal Technology Co., Ltd. (Wuhan, China). Hematoxylin and eosin (H&E) staining kits were obtained from Shanghai Beiou Biotechnology Co., Ltd. (Shanghai, China). All other chemicals and reagents were of analytical grade.

### 2.2. Animals and Experimental Design

This study was conducted in accordance with the National Guidelines for Experimental Animal Welfare (Ministry of Science and Technology, Fuzhouhina, 2006) and was approved by the Laboratory Animal Ethics Committee of Fujian Medical University (Approval No. FJMUIACUC 20190084). A total of 162 specific pathogen-free male BALB/c mice (4–6 weeks old, 20 ± 2 g) were purchased from Shanghai SLAC Laboratory Animal Co., Ltd. (Production License: SCXK (Hu) 20170005). Mice were maintained at 20–22 °C and 60–70% relative humidity under a 12 h light/dark cycle, with free access to standard chow and water. After a 7-day acclimatization period, mice were randomly assigned to six groups (n = 27 per group): normal control group (CK), CTX-induced model group (CTX), low-dose GLPP group (L-GLPP, 50 mg/kg/day), medium-dose GLPP group (M-GLPP, 100 mg/kg/day), high-dose GLPP group (H-GLPP, 200 mg/kg/day), and LMS positive control group (LMS, 40 mg/kg/day). The CTX dose and schedule were selected based on published CTX-induced immunosuppression models and preliminary experiments showing stable immune suppression without mortality. The GLPP doses were selected according to preliminary dose-finding experiments and previous GLPP-related studies, with 200 mg/kg/day showing consistent efficacy without obvious adverse effects. The 42-day intervention period was chosen to allow sufficient immune recovery, gut microbiota remodeling, and intestinal mucosal repair after CTX-induced injury. Except for the CK group, all groups received CTX by intraperitoneal injection at 80 mg/kg/day (0.2 mL per mouse) at 9:30 a.m. for 5 consecutive days to establish the immunosuppressive model; the CK group received an equal volume of normal saline. Model establishment was supported by significantly reduced spleen index (*p* < 0.05) and elevated serum IFN-γ levels (*p* < 0.01) in the CTX group compared with the CK group. During the intervention phase, mice in the GLPP groups received the corresponding doses of GLPP by oral gavage, mice in the LMS group received LMS solution, and mice in the CK and CTX groups received normal saline. Body weight and survival status were monitored daily. After 42 days of intervention, mice were fasted for 24 h and euthanized by cervical dislocation. Cecal tissues, cecal contents, and fecal samples were collected immediately, frozen in liquid nitrogen, and stored at −80 °C for subsequent analyses.

### 2.3. Analysis of Routine Immune Parameters

#### 2.3.1. Immune Organ Indices

At the end of the intervention period (day 42), mice were weighed, and the spleen and thymus were aseptically excised and weighed immediately. The immune organ index was calculated as follows [39]:

Organ index (mg/g) = organ wet weight (mg)/terminal body weight (g).

Each assay was performed using biological samples from the indicated number of mice, with technical triplicates where applicable.

#### 2.3.2. Splenic Lymphocyte Proliferation Assay

Concanavalin A (ConA)-induced splenic lymphocyte proliferation was measured to evaluate cellular immune function. Spleens were aseptically isolated, homogenized in Hank’s balanced salt solution, and filtered through a cell sieve. The cell suspension was centrifuged at 500 rpm for 10 min, and erythrocytes were removed using lysis buffer. Splenic lymphocytes were resuspended in RPMI 1640 medium at 3–5 × 10^6^ cells/mL. A 100 μL aliquot of cell suspension was added to each well of a 96-well plate, with six replicate wells per group. ConA was added at 10 μg/mL, and wells without ConA were used as blank controls. Plates were incubated at 37 °C in a 5% CO_2_ incubator for 48 h, followed by addition of 10% Cell Counting Kit-8 (CCK-8) solution. After 1 h of incubation, absorbance was measured at 450 nm using a microplate reader.

#### 2.3.3. Determination of Delayed-Type Hypersensitivity (DTH) Induced by 2,4-Dinitrofluorobenzene (DNFB)

DNFB was used to establish a DTH model for evaluating antigen-specific cellular immune responses. A 1:1 (*v*/*v*) acetone–sesame oil mixture was used as the solvent, and 50 mg of DNFB was dissolved in the mixture to prepare the sensitization solution. The abdominal hair of mice was removed over an area of approximately 3 × 3 cm, and 50 μL of DNFB solution was evenly applied to the depilated area for sensitization. Four days later, 10 μL of DNFB solution was applied to both sides of the right ear for challenge. At 24 h after challenge, 8-mm circular tissue discs were excised from both ears and weighed immediately. The DTH response was quantified as the weight difference between the right and left ear discs.

#### 2.3.4. Assessment of Macrophage Phagocytic Function by Carbon Clearance Assay

Macrophage phagocytic function was evaluated using the carbon clearance assay. Diluted India ink solution (10 mL/kg) was injected intravenously through the tail vein, and timing was started immediately after injection. At 2 and 10 min after injection, 20 μL of blood was collected from the inner canthus vein and mixed with 2 mL of 0.1% Na_2_CO_3_ solution. Absorbance was measured at 550 nm using a microplate reader, with 0.1% Na_2_CO_3_ solution as the blank control. After blood collection, mice were euthanized, and the liver and spleen were collected and weighed to calculate the carbon clearance index.

#### 2.3.5. Quantification of Immunoglobulin and Cytokine Levels

Retro-orbital blood was collected aseptically and centrifuged at 1000 rpm for 10 min to obtain serum. The concentrations of IgA, IL-2, IFN-γ, and TNF-α were quantified using commercial ELISA kits according to the manufacturers’ protocols.

#### 2.3.6. Histopathological Analysis of Spleen, Thymus, and Intestine

Spleen, thymus, and intestinal tissues were aseptically isolated and fixed in 4% paraformaldehyde for 24 h. The fixed tissues were embedded in paraffin, sectioned into 5 μm slices, and stained with H&E. Histological changes were observed and imaged under an optical microscope (Keyence VHX-7000, Osaka, Japan) at 200× magnification.

### 2.4. 16S rRNA Gene Sequencing and Analysis

Total genomic DNA was extracted from fecal samples using the sodium dodecyl sulfate (SDS)-based lysis method, and DNA purity and concentration were assessed by 2% agarose gel electrophoresis. The V3-V4 hypervariable region of the bacterial 16S rRNA gene was amplified by PCR using primers 515F (5′-GTGCCAGCMGCCGCGGTAA-3′) and 806R (5′-GGACTACHVGGGTWTCTAAT-3′). Purified PCR amplicons were subjected to high-throughput sequencing on the Illumina NovaSeq 6000 platform (Illumina, San Diego, CA, USA).

Raw sequencing data were denoised and quality-filtered using the QIIME pipeline (version 1.9.1) to obtain clean reads [40]. Operational taxonomic units (OTUs) were clustered at 97% sequence similarity using UPARSE (version 7.0.1001) [41]. Taxonomic annotation was performed against the SILVA 138.1 database using the Mothur method with a confidence threshold of 0.8–1.0 [42]. Alpha-diversity indices, including observed species, Chao1, Shannon, and Simpson, were calculated using QIIME, and intergroup differences were analyzed using the Kruskal–Wallis rank-sum test. Beta diversity was evaluated using principal component analysis (PCA), principal coordinate analysis (PCoA), and linear discriminant analysis effect size (LEfSe). Results were visualized using R software (version 3.6.3).

### 2.5. UPLC-QTrap-MS/MS-Based Metabolomic Analysis

Cecal content samples (50 mg) were thawed and homogenized with 500 μL of precooled (−20 °C) 70% methanol aqueous solution containing 1 μM 2-chlorophenylalanine as the internal standard. The mixture was vortexed for 3 min, sonicated in an ice-water bath for 10 min, and centrifuged at 12,000 rpm for 10 min at 4 °C. The supernatant was collected for UPLC-QTrap-MS/MS analysis. A quality control (QC) sample was prepared by pooling 20 μL of supernatant from each experimental sample and was analyzed after every 10 samples to monitor analytical reproducibility.

Chromatographic separation was performed on a Shim-pack UFLC SHIMADZU CBM30A system using an ACQUITY UPLC HSS T3 column (1.8 μm, 2.1 × 100 mm; Waters, Milford, MA, USA) at 40 °C. The mobile phase consisted of 0.1% formic acid in water (phase A) and 0.1% formic acid in acetonitrile (phase B). The gradient elution program was as follows: 0–1 min, 95:5 (A:B, v/v); 1–11 min, linear gradient to 10:90 (v/v); 11–12 min, maintained at 10:90 (v/v); and 12.1–14 min, restored to 95:5 (v/v). The flow rate was 0.4 mL/min, and the injection volume was 2 μL.

Mass spectrometry was performed on a SCIEX QTRAP^®^ mass spectrometer equipped with an electrospray ionization source operating in positive and negative ion switching mode. The ion spray voltage was 5500 V (positive) and −4500 V (negative); source temperature 500 °C; ion source gas I 55 psi; ion source gas II 60 psi; curtain gas 25 psi; and collision gas set to high. Optimized declustering potential and collision energy were applied for each metabolite [43]. Data acquisition was performed using Analyst 1.6.3 software (SCIEX).

Metabolite annotation was performed by matching retention time, precursor ion, and MS/MS spectra against the Metware Database (MWDB) and public databases, including the Human Metabolome Database (HMDB), the METLIN Metabolite Database (METLIN), and the Kyoto Encyclopedia of Genes and Genomes (KEGG). According to the Metabolomics Standards Initiative (MSI) guidelines, all reported metabolites are classified as putative annotations (MSI Level 2). Lipids were annotated following Lipid MAPS recommendations.

Chromatographic peak integration and calibration were performed using MultiQuant software (version 3.0.1, SCIEX) to generate a multivariate data matrix containing retention time (Rt), mass-to-charge ratio (m/z), and ion intensity. Features with >80% missing values were excluded, and missing values were imputed using half of the minimum positive value. Data were normalized by Z-score standardization (mean-centered and scaled to unit variance). Features with relative standard deviation >30% in QC samples were excluded. Metabolomic analysis was performed on six biological replicates per group (n = 6).

Processed data were imported into SIMCA-P 14.0 for unsupervised principal component analysis (PCA) to visualize overall metabolic trends, and supervised partial least squares–discriminant analysis (PLS-DA) was used as a complementary visualization tool for group separation. Differential metabolites were selected based on univariate analysis (independent-sample *t*-test, *p* < 0.05), fold change (FC ≥ 2 or FC ≤ 0.5), and variable importance in projection (VIP > 1). Metabolic pathway enrichment analysis was performed using MetaboAnalyst 5.0. Visualization was performed using R software (version 3.6.3).

### 2.6. Transcriptome Analysis

Total RNA was extracted from mouse cecal tissues using the NEB RNA Extraction Kit (New England Biolabs, Beijing, China) according to the manufacturer’s protocol. RNA concentration and purity were measured using a NanoDrop ND2000 spectrophotometer (Thermo Fisher Scientific, Waltham, MA, USA), and RNA integrity was assessed by agarose gel electrophoresis. Samples with RNA integrity number values greater than 7 were used for sequencing library construction. Libraries were prepared using the NEBNext Ultra RNA Library Prep Kit for Illumina and sequenced on the Illumina HiSeq platform.

Raw reads were filtered using fastp to obtain clean reads, which were then aligned to the mouse mm10 reference genome using HISAT2. Gene expression levels were quantified as fragments per kilobase of transcript per million mapped reads (FPKM). Reproducibility among biological replicates was evaluated using Pearson correlation coefficients (R^2^ > 0.9). Differential expression analysis was performed using DESeq2 with the criteria of |log_2_FoldChange| > 1 and false discovery rate (FDR) < 0.05 [44,45]. KEGG pathway enrichment analysis was conducted to annotate the biological functions of differentially expressed genes (DEGs).

### 2.7. Statistical Analysis

Data were analyzed using Excel, R software (version 3.6.3), and SPSS 19.0 (IBM Corp., Armonk, NY, USA), and are expressed as the mean ± standard deviation (SD). Graphical representations were generated using GraphPad Prism 8.0 (GraphPad Software, San Diego, CA, USA). One-way analysis of variance (ANOVA) followed by Duncan’s multiple range test was used to determine intergroup differences. A value of *p* < 0.05 was considered statistically significant, and *p* < 0.001 was considered highly significant. Normality was assessed using the Shapiro–Wilk test. For data with skewed distribution or unequal variances, the Kruskal–Wallis rank-sum test was applied. No samples were excluded as outliers. The Mantel test was used to evaluate associations between gut microbiota and host metabolites at different taxonomic levels. Pearson correlation analysis was used to construct the correlation network between differential metabolites and DEGs. Multi-omics data integration and visualization were performed using R packages including vegan, pheatmap, and igraph. Correlation-based results were interpreted as associations rather than direct evidence of causality.

## 3. Results and Discussion

### 3.1. GLPP Ameliorates CTX-Induced Pathological Injury in the Intestinal Tract and Immune Organs

**Figure 1:**
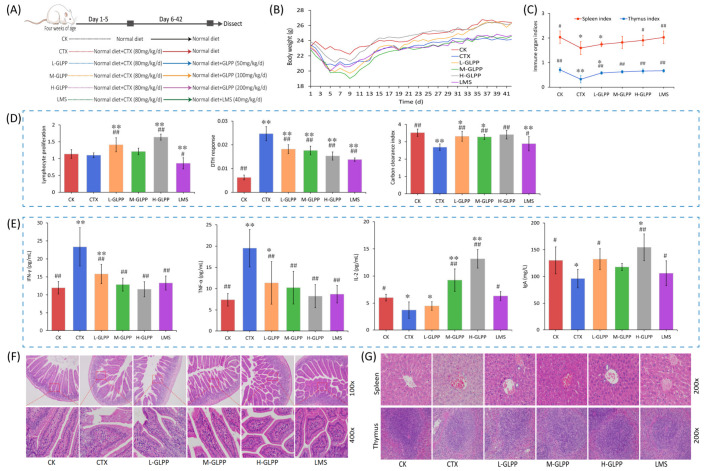
Effects of GLPP on CTX-induced immunosuppression and intestinal mucosal injury in mice. (**A**) Experimental schedule; (**B**) body weight changes; (**C**) immune organ indices, including spleen and thymus indices; (**D**) splenic lymphocyte proliferation, DNFB-induced delayed-type hypersensitivity, and carbon clearance index; (**E**) serum immune factors, including IFN-γ, IL-2, TNF-α, and IgA; (**F**) representative H&E-stained intestinal sections; (**G**) representative H&E-stained spleen and thymus sections. Data are presented as mean ± SD (Compared with CK, *, *p* < 0.05, **, *p* < 0.01; compared with CTX, #, *p* < 0.05, ##, *p* < 0.01).

CTX is a broad-spectrum alkylating agent widely used for the treatment of malignant tumors and autoimmune diseases [46]. However, its non-specific cytotoxicity can cause adverse effects such as myelosuppression, gastrointestinal mucosal injury, and immunosuppression [47,48,49]. In the present study, a CTX-induced mouse model characterized by immunosuppression and intestinal mucosal injury was established to evaluate the protective effects of GLPP (Figure 1A). Dynamic monitoring of body weight revealed phase-dependent changes among the experimental groups (Figure 1B). All CTX-treated groups showed an initial decrease in body weight, which may be attributed to CTX-induced gastro-intestinal toxicity, impaired nutrient absorption, and metabolic disturbance. The CTX model group exhibited the most pronounced and persistent weight loss, whereas GLPP-treated mice showed accelerated body-weight recovery in a dose-dependent manner. At the experimental endpoint, no significant difference in body weight was observed between the H-GLPP and CK groups, suggesting that GLPP alleviated CTX-associated metabolic disturbance and promoted recovery.

CTX administration significantly impaired immune organ development, as reflected by reduced spleen index (1.592 ± 0.266 mg/g vs. 2.030 ± 0.253 mg/g in CK, *p* < 0.01) and thymus index (0.322 ± 0.146 mg/g vs. 0.703 ± 0.091 mg/g in CK, *p* < 0.01). GLPP intervention restored these immune organ indices in a dose-dependent manner. In the H-GLPP group, the spleen index increased to 1.902 ± 0.212 mg/g (*p* < 0.01 vs. CTX) and the thymus index increased to 0.656 ± 0.081 mg/g (*p* < 0.01 vs. CTX), reaching levels close to the CK and LMS groups (Figure 1C).

The systemic immunomodulatory effects of GLPP were further evaluated using splenic lymphocyte proliferation, DTH, and carbon clearance assays (Figure 1D). No significant difference in splenic lymphocyte proliferation was observed between the CTX and CK groups, suggesting that CTX-induced immune dysfunction may involve immune organ impairment rather than direct suppression of splenic lymphocyte viability under the present assay conditions. GLPP intervention significantly enhanced lymphocyte proliferation in a dose-dependent manner. Compared with the CTX group (OD_450_ = 1.065 ± 0.051), the H-GLPP group showed a 1.44-fold increase in proliferation activity (OD_450_ = 1.529 ± 0.073, *p* < 0.01), which was also significantly higher than the LMS group (OD_450_ = 0.834 ± 0.145, *p* < 0.01). In the DTH assay, CTX significantly suppressed the DNFB-induced ear swelling response (0.0060 ± 0.0011 vs. 0.0185 ± 0.0022 in CK, *p* < 0.01). GLPP dose-dependently normalized the response; the H-GLPP group showed an ear swelling of 0.0243 ± 0.0031 (*p* < 0.01 vs. CTX), which was 4.05-fold higher than the CTX group and significantly greater than the LMS group (0.0139 ± 0.0008, *p* < 0.01). In addition, H-GLPP significantly increased the carbon clearance index α from 2.650 ± 0.164 in the CTX group to 3.403 ± 0.223 (*p* < 0.01), close to the CK level (3.522 ± 0.207), indicating enhanced macrophage phagocytic activity. Together, these results suggest that GLPP improves both adaptive immune responses and innate macrophage-related immune function.

Compared with the CK group (IFN-γ: 11.90 ± 1.91 pg/mL; TNF-α: 7.09 ± 1.34 pg/mL), the CTX group showed significantly elevated levels of IFN-γ (21.91 ± 4.20 pg/mL, *p* < 0.01) and TNF-α (19.48 ± 4.64 pg/mL, *p* < 0.01), indicating an abnormal inflammatory state following CTX exposure. GLPP intervention dose-dependently reduced these excessive pro-inflammatory cytokine levels. In the H-GLPP group, IFN-γ decreased to 12.07 ± 1.81 pg/mL (*p* < 0.01 vs. CTX) and TNF-α decreased to 7.93 ± 2.92 pg/mL (*p* < 0.01 vs. CTX), both reaching levels comparable to the CK group. Meanwhile, IL-2 levels increased from 3.99 ± 1.66 pg/mL in the CTX group to 13.13 ± 1.89 pg/mL in the H-GLPP group (*p* < 0.01), representing a 3.29-fold increase, with the H-GLPP group showing the highest IL-2 level among all groups. The M-GLPP group (8.92 ± 2.08 pg/mL, *p* < 0.01 vs. CTX) and LMS group (6.34 ± 0.90 pg/mL, *p* < 0.05 vs. CTX) also showed significantly elevated IL-2 levels compared with the CTX group. GLPP also restored IgA levels. Compared with the CTX group (96.282 ± 17.820 mg/L), the H-GLPP group showed significantly elevated IgA (154.621 ± 24.821 mg/L, *p* < 0.01), which was also significantly higher than the LMS group (106.275 ± 22.322 mg/L, *p* < 0.05). These findings indicate that GLPP attenuated excessive inflammatory responses while supporting T cell-associated and IgA-related immune function.

Histopathological analysis further confirmed the protective effects of GLPP on the intestine and immune organs (Figure 1F,G). The CK group exhibited intact intestinal mucosa, polarized epithelial cells, and uniformly arranged villi. In contrast, the CTX group showed severe intestinal mucosal injury, including loss of epithelial polarity, cellular edema, and extensive villus disruption. GLPP intervention, especially at the high dose, markedly alleviated these pathological alterations, as evidenced by improved villus structure and reduced epithelial shedding. In immune organs, CTX treatment induced splenic white pulp lymphoid follicle atrophy, red pulp reticular fiber disruption, thymic cortex thinning, and lymphocyte depletion. GLPP administration restored splenic lymphoid follicles and increased lymphocyte density in the thymic cortex, with the H-GLPP group showing tissue morphology close to that of the CK group.

Overall, GLPP counteracted CTX-induced immunosuppression and intestinal mucosal injury. High-dose GLPP reduced body-weight loss, restored thymus and spleen structure and indices, enhanced lymphocyte proliferation and macrophage phagocytic activity, and normalized immune-related cytokine and IgA responses. Compared with LMS, GLPP showed comparable or stronger immuno-modulatory and gut-protective effects in several assays. These findings support the potential of GLPP as a food-derived bioactive ingredient for mitigating CTX-associated immune and intestinal disturbances and enhancing immune resilience.

### 3.2. Effects of GLPP on the Fecal Gut Microbiota of CTX-Immunosuppressed Mice

**Figure 2:**
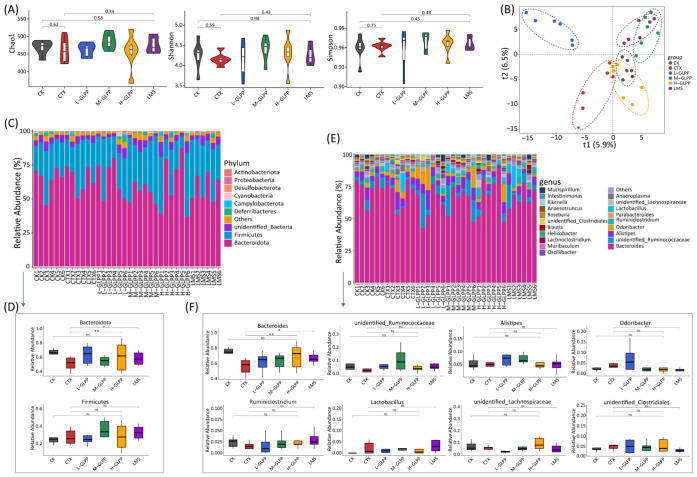
Effects of GLPP on fecal gut microbiota structure in CTX-induced immunosuppressed mice. (**A**) Alpha-diversity indices, including Chao1, Simpson, and Shannon; (**B**) beta-diversity analysis based on PCA; (**C**) microbial composition at the phylum level; (**D**) relative abundance of dominant bacterial phyla, including Bacteroidota and Firmicutes; (**E**) microbial composition at the genus level; (**F**) relative abundance of representative genera. Statistical significance was determined by one‑way ANOVA followed by Duncan’s multiple range test (* *p* < 0.05 and ** *p* < 0.01 indicate significant differences between the groups connected by horizontal lines; ns denotes not significant).

Alpha-diversity indices, including Chao1, Shannon, and Simpson, were used to evaluate the richness and evenness of the fecal gut microbial community [50]. Compared with the CK group (Chao1: 438.874 ± 56.168; Shannon: 6.128 ± 0.535), the CTX group exhibited reduced Chao1 (404.555 ± 21.094, *p* < 0.05) and Shannon (5.996 ± 0.242, *p* > 0.05) values. GLPP intervention increased alpha diversity in a dose-dependent manner. In the H-GLPP group, Chao1 increased to 410.369 ± 46.244 and Shannon to 6.199 ± 0.642 (Figure 2A). Beta-diversity analysis revealed clear separation between the CK and CTX groups (Figure 2B), confirming that CTX induced gut microbial dysbiosis. GLPP intervention shifted the microbial community structure toward that of the CK group, suggesting that GLPP ameliorated CTX-induced ecological disturbance in the gut.

At the phylum level, Bacteroidota, Firmicutes, and unidentified Bacteria were the dominant phyla across groups (Figure 2C), with significant intergroup differences in relative abundance. CTX treatment reduced the relative abundance of Bacteroidota compared with the CK group (0.642 ± 0.100 vs. 0.641 ± 0.095, *p* > 0.05), whereas H-GLPP (0.611 ± 0.214) and LMS (0.579 ± 0.095) showed a trend toward restoration (Figure 2D). No significant difference in Firmicutes abundance was observed among groups. Because Bacteroidota is closely associated with host metabolism and immune regulation [51,52], its restoration may contribute to the recovery of metabolic and immune homeostasis.

At the genus level, the dominant taxa included *Bacteroides*, unidentified_Ruminococcaceae, *Alistipes*, and *Odoribacter* (Figure 2E). CTX treatment decreased the relative abundances of *Bacteroides*, unidentified_Ruminococcaceae, and *Ruminiclostridium*, while increasing the abundances of *Alistipes* and unidentified_Lachnospiraceae. GLPP intervention, particularly H-GLPP, increased the abundances of *Bacteroides*, unidentified_Ruminococcaceae, and *Ruminiclostridium* and reduced the abundance of *Alistipes*. Increased abundance of *Alistipes* and some Lachnospiraceae-related taxa has been associated in certain contexts with inflammatory or metabolic disturbances, although their functions may be host- and condition-dependent. *Bacteroides* showed the most pronounced intergroup variation, with the highest relative abundance observed in the H-GLPP group (Figure 2E,F). *Bacteroides* and *Ruminiclostridium* are important members of the gut microbial ecosystem and participate in polysaccharide utilization, short-chain fatty acid production, immune regulation, and metabolic balance [53,54]. These results suggest that H-GLPP improved gut microbial homeostasis by enriching potentially beneficial taxa and suppressing taxa associated with dysbiosis.

**Figure 3:**
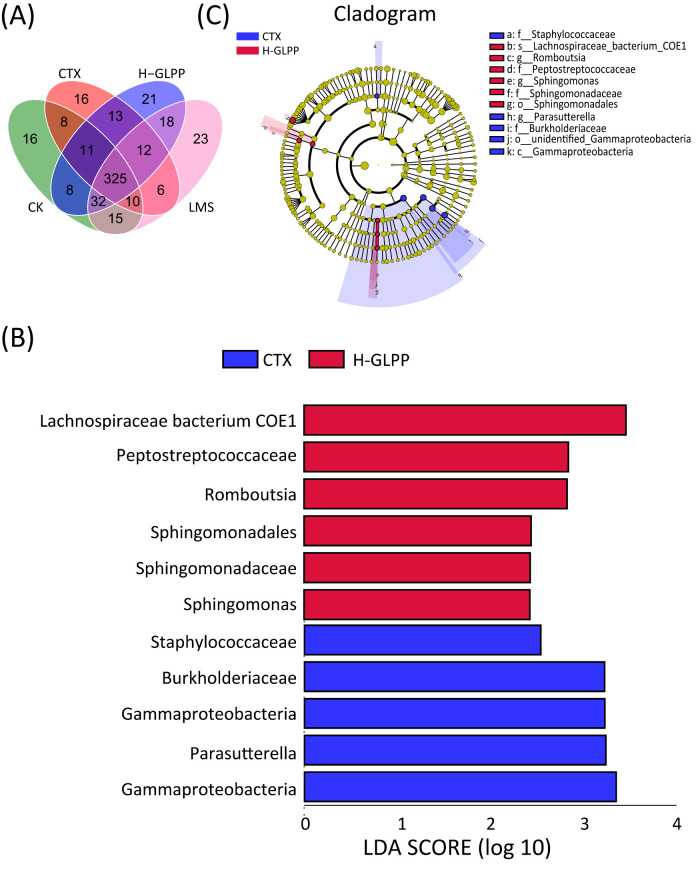
(**A**) Venn analysis of shared and unique OTUs; (**B**) LEfSe cladogram; (**C**) LDA score plot of differentially abundant taxa.

Venn diagram analysis identified 325 shared OTUs across all groups, with 23, 21, 16, and 16 unique OTUs in the CTX, L-GLPP, M-GLPP, and H-GLPP groups, respectively (Figure 3A). These group-specific OTUs indicate that CTX and GLPP treatments were associated with distinct microbial signatures. LEfSe analysis (LDA score > 1) was used to identify differentially abundant microbial taxa among groups (Figure 3B,C; Table 1). The H-GLPP group was enriched in taxa including *Lachnospiraceae_bacterium_COE1*, *Romboutsia*, Peptostreptococcaceae, and Sphingomonadales, whereas the CTX group was enriched in Staphylococcaceae, *Parasutterella*, Burkholderiaceae, unidentified_Gammaproteobacteria, and Gammaproteobacteria. These results further confirm that GLPP reshaped the gut microbial community in CTX-treated mice. For certain taxa, the standard deviation exceeded the mean (e.g., *Bifidobacterium* in LMS, *Bacteroides* in CTX, and *Odoribacter* in L-GLPP; Table 1), indicating a skewed distribution driven by a few high-abundance samples. The median and interquartile range (IQR) calculated from the original OTU table (Table S1) confirmed the same directional trends. Inter-group comparisons were performed using the Kruskal–Wallis non-parametric test to minimize the influence of outliers, and no samples were excluded.

In conclusion, H-GLPP restored intestinal microbial diversity, enriched potentially beneficial microbes such as *Bacteroides*, unidentified_Ruminococcaceae, and *Ruminiclostridium*, and reduced dysbiosis-associated taxa such as *Alistipes*. These findings are consistent with previous studies showing that *G. lucidum* polysaccharides modulate the gut microbiota and regulate immune cell function to alleviate intestinal inflammation [55], as well as reports that mushroom-derived polysaccharides exert anti-inflammatory and immunomodulatory activities [56]. Similar to the regulatory effects of *G. lucidum* β-D-glucans with different molecular weights in ulcerative colitis models [57], GLPP-mediated gut microbiota remodeling may represent an important link in its immunomodulatory effects. These microbial changes provide a biological basis for the subsequent modulation of host metabolic and transcriptional profiles, which is further supported by integrated foodomics correlation analysis in Section 3.5.

### 3.3. GLPP Modulates Cecal Metabolites in CTX-Induced Immunosuppressed Mice

**Figure 4:**
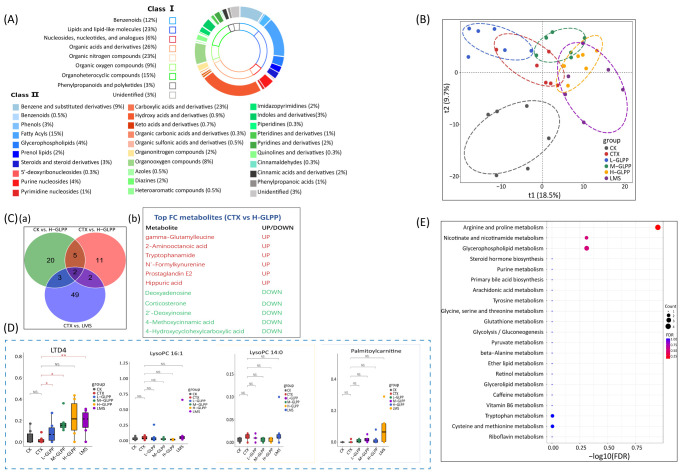
Effects of GLPP on cecal metabolites in CTX-induced immunosuppressed mice. (**A**) Sunburst plot showing biochemical classification of identified metabolites; (**B**) PLS-DA score plot; (**C**) Venn analysis and representative differential metabolites; (**D**) shared differential metabolites between H-GLPP and LMS groups; (**E**) KEGG pathway enrichment analysis of differential metabolites (* *p* < 0.05 and ** *p* < 0.01 indicate significant differences between the groups connected by horizontal lines; NS denotes not significant).

A broadly targeted metabolomics approach based on UPLC-QTrap-MS/MS was used to investigate changes in small-molecule metabolites in cecal contents from CTX-induced immunosuppressed mice. Metabolite classification was visualized using a sunburst chart (Figure 4A), which identified eight major categories: organic acids and derivatives (26%), organic nitrogen compounds (23%), lipids and lipid-like molecules (23%), organoheterocyclic compounds (15%), benzenoids (12%), organic oxygen compounds (9%), nucleosides/nucleotides and analogues (6%), and others (3%). Twenty-nine secondary metabolite subclasses were also identified, mainly including carboxylic acids and derivatives, fatty acyls, benzene and substituted derivatives, and organooxygen compounds. PLS-DA showed distinct clustering patterns among groups, with the H-GLPP and LMS groups showing partially overlapping metabolic profiles (Figure 4B), suggesting that H-GLPP shifted the CTX-disturbed metabolome toward a profile similar to that of the positive control.

A total of 92 differential metabolites were putatively annotated across the compared groups (Table 2). The LMS vs. CTX comparison showed the largest difference, with 56 differential metabolites (30 upregulated and 26 downregulated), followed by the H-GLPP vs. CTX comparison with 20 differential metabolites (12 upregulated and 8 downregulated). Venn analysis further revealed group-specific and shared metabolic changes (Figure 4C). In the CTX vs. H-GLPP comparison, 11 distinct differential metabolites were putatively annotated, including gamma-Glutamylleucine(γ-Glu-Leu), 2-aminooctanoic acid, prostaglandin E2, and hippuric acid. These metabolites are associated with amino acid metabolism, inflammatory regulation, and microbial–host co-metabolism, suggesting that GLPP may contribute to immune recovery by remodeling intestinal metabolic pathways.

Four shared differential metabolites, leukotriene D4 (LTD4), LysoPC 16:1, LysoPC 14:0, and palmitoylcarnitine, were identified between the H-GLPP and LMS groups (Figure 4D). LTD4 levels were reduced in the CTX group compared with the CK group, whereas GLPP and LMS interventions increased LTD4 levels relative to the CTX group (*p* < 0.05). These results suggest that GLPP may influence lipid mediator profiles, although the biological significance of these alterations requires further experimental validation. No significant changes in LysoPC 16:1, LysoPC 14:0, or palmitoylcarnitine concentrations were observed across the treatment groups.

Peak intensity normalization and hierarchical clustering analysis were performed to further evaluate the effect of GLPP on intestinal metabolites (Figure S3A). GLPP and LMS administration increased the levels of corticosterone, theobromine, 2-deoxyinosine, and deoxyadenosine. Corticosterone is a well-characterized immunoregulatory hormone that limits excessive leukocyte accumulation and prevents tissue damage [58]. The observed changes in corticosterone-related metabolites suggest a potential association with GLPP intervention, warranting further investigation.

Pearson correlation analysis of 56 differential metabolites between the CTX and LMS groups revealed significant positive correlations between LysoPC 14:0 and multiple lipid derivatives, including LysoPC 16:0, 16:1, 18:0, 18:1, 20:1, and 20:2, as well as energy-related metabolites such as creatine and lactic acid (Figure S3B). In contrast, N-acetylglucosamine 1-phosphate and ADP-ribose were negatively correlated with these LysoPC species. These metabolites are closely related to lipid remodeling, energy metabolism, and cellular biosynthetic activity, suggesting that LMS and GLPP may regulate host metabolic status through overlapping lipid- and energy-related pathways.

KEGG pathway enrichment analysis of 56 differential metabolites between the CTX and LMS groups identified three significantly enriched metabolic pathways: arginine–proline metabolism, nicotinate and nicotinamide metabolism, and glycerophospholipid metabolism (Figure 4E). These pathways are associated with amino acid metabolism, vitamin-derived cofactor metabolism, membrane lipid remodeling, and immune cell function.

Together, these findings indicate that GLPP is associated with alterations in metabolic pathways related to amino acid metabolism, nicotinate and nicotinamide metabolism, and glycerophospholipid metabolism, as well as changes in key metabolites including gamma-Glutamylleucine, 2-aminooctanoic acid, LTD4, and corticosterone. Similar to the protective effects of polysaccharide peptides against chemotherapy-induced intestinal injury [59], GLPP may alleviate CTX-induced toxicity through metabolic regulation. The differential metabolites putatively annotated in this study, particularly LTD4, gamma-Glutamylleucine, and hippuric acid, may represent candidate metabolic mediators potentially linking GLPP-associated gut microbiota remodeling with host immune regulation. These findings are further supported by transcriptomic and integrated foodomics correlation analyses.

### 3.4. Effects of GLPP on the Cecal Transcriptome of CTX-Immunosuppressed Mice

To elucidate the molecular mechanisms associated with GLPP-mediated immunomodulation at the transcriptional level, transcriptome sequencing was performed on mouse cecal tissues, with a focus on pattern recognition receptors (PRRs) and downstream immune signaling pathways. Hierarchical clustering analysis (Figure S4) revealed distinct intergroup differences in gene expression profiles, with clear separation among experimental groups.

**Figure 5:**
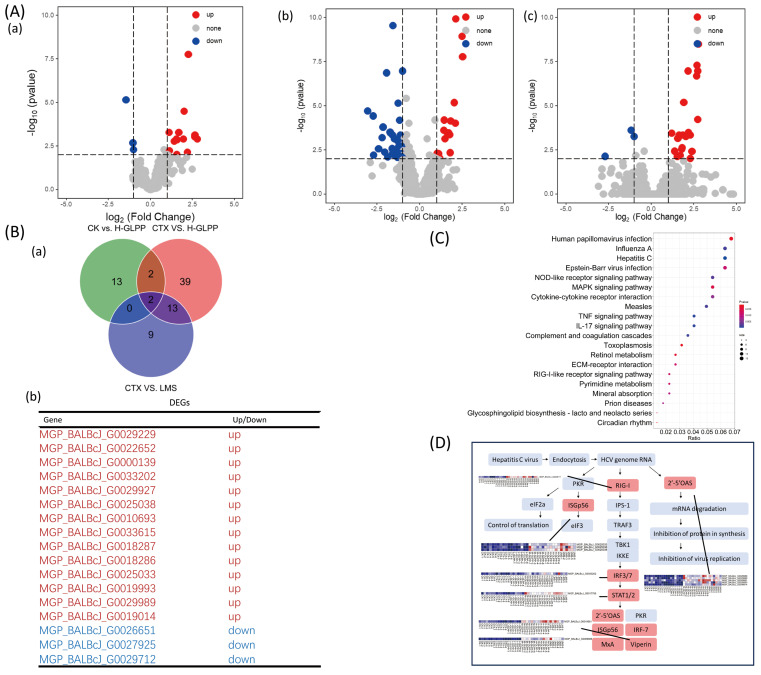
Transcriptomic landscape and functional annotation of GLPP-modulated genes in CTX-induced immunosuppressed mice. (**A**) Volcano plots of DEGs in CK vs. H-GLPP, CTX vs. H-GLPP, and CTX vs. LMS comparisons; (**B**) Venn analysis of shared and group-specific DEGs; (**C**) KEGG pathway enrichment analysis; (**D**) PRR-related immune signaling network associated with GLPP-mediated immune regulation.

Volcano plot analysis identified DEGs in the CK vs. H-GLPP, CTX vs. H-GLPP, and CTX vs. LMS comparisons: 17 DEGs (14 upregulated and 3 downregulated), 56 DEGs (18 upregulated and 38 downregulated), and 24 DEGs (21 upregulated and 3 downregulated), respectively (Figure 5A). Venn diagram analysis showed 17 overlapping DEGs, as well as 39, 13, and 9 group-specific DEGs (Figure 5B). Several PRR-related genes, including RIG-I, PKR, and genes associated with NOD-like receptor signaling, were among the DEGs, suggesting that PRR-related signaling may participate in GLPP-associated immune regulation.

KEGG pathway enrichment analysis was conducted to annotate the biological functions of DEGs (Figure 5C). DEGs from the CTX vs. H-GLPP and CTX vs. LMS comparisons were predominantly enriched in pathways annotated as human papillomavirus infection and influenza A (Figures S1 and S2). Although these pathways are categorized as viral infection-related, they contain many genes involved in innate immune recognition, PRR activation, interferon signaling, and cytokine regulation. Therefore, their enrichment should be interpreted as evidence that GLPP modulates host immune defense signaling rather than as direct antiviral activity, consistent with the known crosstalk between PRR signaling and metabolic pathways in innate immunity [60,61]. In the CK vs. H-GLPP comparison, DEGs were also enriched in immune-related pathways including NOD-like receptor signaling, mitogen-activated protein kinase (MAPK) signaling, and cytokine–cytokine receptor interaction.

Further analysis showed that key downstream genes associated with PRR signaling, including RIG-I, 2′-5′OAS, ISGp56, IRF3/7, and STAT1/2, were upregulated in both the GLPP and LMS groups (Figure 5D). These transcriptomic changes suggest activation of PRR-associated intracellular immune signaling. The upregulated immune-related genes may contribute to the macrophage and lymphocyte functional responses observed in this study and were correlated with key immunoregulatory metabolites modulated by GLPP, supporting the presence of a metabolite–gene–immune regulatory axis.

Transcriptomic results also suggested crosstalk between PRR-related signaling and the arginine–proline metabolic pathway identified by metabolomic profiling. Previous studies have shown that arginine–proline metabolism plays important roles in immune function and interacts with PRR signaling through nitric oxide synthase/arginase pathways [62,63]. In the present study, GLPP-associated changes in PRR-related genes were accompanied by changes in genes involved in Arg-Pro metabolism, including ARG1, ODC, and PRODH. These findings suggest that PRR/MAPK/nuclear factor kappa B (NF-κB)-related signaling may interact with amino acid metabolic reprogramming during GLPP-mediated immune restoration.

Physiologically, CTX-induced immunosuppression was accompanied by reduced expression of PRR-related genes compared with the CK group. High-dose GLPP reversed this trend and restored PRR-related gene expression toward normal levels, which may contribute to the recovery of Arg-Pro metabolic homeostasis observed in metabolomic profiling. Potential synergy may exist between GLPP-associated gut microbiota remodeling and host PRR signaling: GLPP may reduce dysbiosis-associated microbial signals and increase beneficial metabolites such as short-chain fatty acids and γ-Glu-Leu, which may in turn regulate PRR expression and downstream immune activity. This interpretation requires further validation using pathway inhibition or microbiota intervention experiments.

Collectively, transcriptomic analysis suggested that PRR-mediated signaling and its interaction with Arg-Pro metabolism are important molecular pathways associated with the protective effects of GLPP against CTX-induced immunosuppression. These findings provide gene-level evidence supporting a gut microbiota–metabolite–PRR–immune regulatory axis and are consistent with the integrated foodomics conclusions of this study.

### 3.5. Integrated Foodomics Correlation Analysis

**Figure 6:**
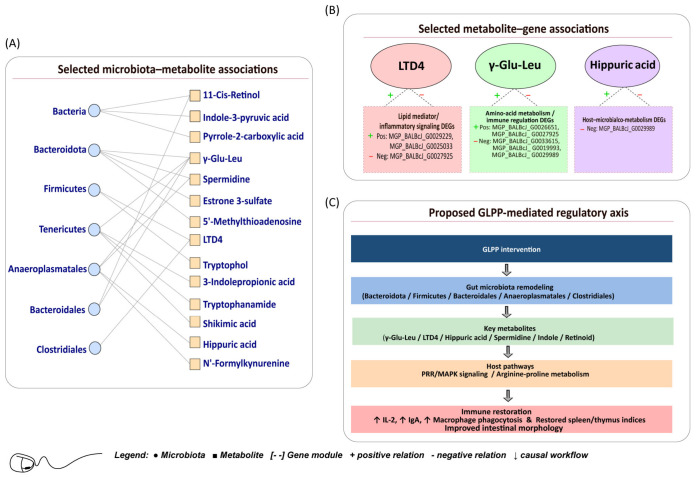
Simplified integrated foodomics network showing key microbiota–metabolite–gene associations associated with GLPP-mediated immune restoration. (**A**) Selected microbiota–metabolite associations. (**B**) Filtered metabolite–gene correlation network. Edge color indicates positive or negative correlation. (**C**) Proposed GLPP-mediated gut microbiota–metabolite–gene–immune regulatory axis.

Integrated foodomics analysis, including microbiomics, metabolomics, and transcriptomics, was performed to delineate the regulatory network associated with GLPP-mediated immune and metabolic remodeling in immunosuppressed mice. The full Mantel test maps and complete metabolite-DEG correlation matrices are provided in Supplementary Figure S5A–D, while the main figure presents a simplified summary of the key associations (Figure 6A–C).

As summarized in Figure 6A, GLPP-responsive microbial taxa, including Bacteroidota, Firmicutes, Tenericutes, Bacteroidales, Anaeroplasmatales, and Clostridiales, showed associations with key immunologically active metabolites. Among these, 11-cis-retinol, indole-3-pyruvic acid, and pyrrole-2-carboxylic acid were associated with the overall bacterial community. Bacteroidota showed associations with γ-Glu-Leu, spermidine, estrone 3-sulfate, and 5′-deoxy-5′-(methylthio)adenosine; Firmicutes was associated with LTD4 and tryptophol; Tenericutes was associated with γ-Glu-Leu, 3-indolepropionic acid, tryptophanamide, and shikimic acid. At the order level, Anaeroplasmatales was associated with γ-Glu-Leu, hippuric acid, N′-formylkynurenine, and spermidine; Bacteroidales with γ-Glu-Leu and 11-cis-retinol; and Clostridiales with LTD4. These associations suggest that GLPP-mediated gut microbiota remodeling may influence host metabolism through amino acid metabolism, lipid mediator signaling, tryptophan metabolism, and host–microbial co-metabolism pathways.

To connect these metabolic changes with host transcriptional responses, we constructed a filtered metabolite–gene correlation network (Figure 6B). Key metabolites, including LTD4, γ-Glu-Leu, prostaglandin E2, and hippuric acid, were correlated with DEG modules involved in lipid mediator/inflammatory signaling, amino-acid metabolism/immune regulation, and host–microbial co-metabolism. Specifically, LTD4 showed a negative correlation with MGP_BALBcJ_G0027925 and positive correlations with multiple DEGs, including MGP_BALBcJ_G0029229 and MGP_BALBcJ_G0025033. γ-Glu-Leu displayed bidirectional correlations: negative with MGP_BALBcJ_G0033615, MGP_BALBcJ_G0019993, and MGP_BALBcJ_G0029989; positive with MGP_BALBcJ_G0026651 and MGP_BALBcJ_G0027925. Hippuric acid showed a negative correlation with MGP_BALBcJ_G0029989.

These key metabolites may regulate macrophage and lymphocyte functions through arginine–proline, nicotinate–nicotinamide, and glycerophospholipid metabolic pathways. Nicotinamide adenine dinucleotide (NAD^+^) metabolism, a major branch of nicotinate–nicotinamide metabolism, plays an important role in immune response and inflammatory homeostasis [62,63,64]. Arginine and proline are functional amino acids involved in the synthesis of polyamines and nitric oxide, thereby regulating gene expression, protein phosphorylation, and immune cell activation [62,63,65]. γ-Glu-Leu, one of the key differential metabolites identified in this study, has been reported to possess anti-inflammatory activity [66]. Its bidirectional correlations with immune-related genes further suggest that γ-Glu-Leu may be an important metabolic mediator linking GLPP intervention with immune regulation.

Based on these integrated findings, we propose a GLPP-mediated gut microbiota–metabolite–gene–immune regulatory axis (Figure 6C): GLPP reshapes the gut microbial community, which in turn regulates immunologically relevant metabolites, modulates host immune-related pathways (including PRR/MAPK signaling, arginine–proline metabolism, nicotinate–nicotinamide metabolism, and glycerophospholipid metabolism), and ultimately contributes to immune restoration, as evidenced by improved immune organ indices, cytokine production, and intestinal morphology. Overall, the integrated foodomics network suggests that GLPP restores immune homeostasis through coordinated regulation of gut microbiota, immunoregulatory metabolites, and host immune-related genes. Because these analyses are correlation-based, the associations should be interpreted as mechanistic hypotheses rather than direct evidence of causality.

### 3.6. Functional Food Relevance, Limitations, and Application Potential of GLPP

As a food-derived polysaccharide peptide from the edible and medicinal fungus *G. lucidum*, GLPP has potential as an immune-supporting functional food ingredient. The present study shows that GLPP improved immune organ indices, intestinal mucosal morphology, IgA levels, gut microbiota composition, and host metabolic regulation in CTX-treated mice. These multi-level effects suggest that GLPP may act not as a single-target immune stimulant but as a gut-centered regulator of immune homeostasis. This property is particularly relevant for functional food development because dietary macromolecules often exert moderate but broad biological effects through the intestinal ecosystem.

Several issues should be addressed before GLPP can be translated into functional food products. The processing stability of GLPP during heating, storage, and food-matrix formulation should be evaluated, and its gastrointestinal digestion behavior, bio-accessibility, and fermentability by gut microbiota should be investigated using in vitro digestion and colonic fermentation models. Sensory compatibility, long-term safety, allergenicity, and interactions with chemotherapy agents also require systematic assessment. Although GLPP showed immunoprotective effects in mice, human-equivalent intake levels, safety margins, and long-term dietary effects should be validated in clinical or population-based studies. Because only male mice were used to reduce variability associated with the estrous cycle, future studies should include both sexes to confirm the generalizability of GLPP-mediated immunoprotective effects.

Using conventional body surface area conversion, the high GLPP dose used in mice (200 mg/kg/day) corresponds to an approximate human-equivalent dose of 16.3 mg/kg/day, or about 1.0 g/day for a 60 kg adult. This estimated intake range may be feasible for dietary supplement or functional food formulation, but it should be interpreted cautiously because human pharmacokinetic, safety, and efficacy data are not yet available.

Although GLPP improved body-weight recovery and no obvious adverse effects were observed during the present intervention period, comprehensive safety evaluation remains necessary before long-term dietary application. Future studies should include liver and kidney function, hematological parameters, long-term toxicity, processing stability, and human intervention trials.

## 4. Conclusions

A CTX-induced immunosuppressed mouse model accompanied by intestinal mucosal injury was established to evaluate the protective effects of GLPP. An integrated foodomics strategy combining microbiomics, metabolomics, and transcriptomics was used to explore the multi-target mechanisms of this food-derived bioactive component.

High-dose GLPP (H-GLPP, 200 mg/kg/day) restored spleen and thymus indices, improved adaptive and innate immune responses by enhancing lymphocyte proliferation, DTH responses, and macrophage phagocytosis, and alleviated CTX-induced intestinal mucosal injury by promoting villus structural recovery and reducing epithelial shedding. GLPP also regulated immune-related factors, including IL-2 and IgA, and reduced excessive inflammatory cytokine responses.

At the mechanistic level, GLPP reshaped the gut microbial community, notably taxa related to Bacteroidota, Firmicutes, *Bacteroides*, and *Ruminococcaceae*, and was associated with changes in key immunoregulatory metabolites, including LTD4, γ-Glu-Leu, and hippuric acid. These metabolites were correlated with immune-related genes such as RIG-I, PKR, and 2′-5′OAS and with pathways involving arginine–proline and nicotinate–nicotinamide metabolism, forming a microbiota–metabolite–gene–immune interaction network. Compared with the positive control LMS, H-GLPP showed comparable or stronger efficacy in restoring immune function and regulating inflammatory responses in CTX-treated mice.

Collectively, these findings suggest that GLPP restores immune homeostasis through a gut microbiota–metabolite–gene–immune axis. As a natural polysaccharide–peptide conjugate from the edible and medicinal fungus *G. lucidum*, GLPP represents a promising functional ingredient for immune-supporting food products. However, further experimental validation is required to confirm the causal relationships suggested by these correlation-based findings, as well as to assess its processing stability, gastrointestinal behavior, long-term safety, effective human intake, and clinical efficacy.

## Figures and Tables

**Table 1:** Relative abundance and statistical analysis of differential gut microorganisms in BALB/c mice with GLPP and LMS intervention.

No.	Phylum	Order	Genus	Species	Relative Abundance (×10^3^)						*p* Value	LDA Score
					CK	CTX	L-GLPP	M-GLPP	H-GLPP	LMS		
1	Actinobacteria	/	/	/	0.071 ± 0.100	0.027 ± 0.065↓	0.018 ± 0.032↓	0.084 ± 0.125↑	0.075 ± 0.147↑	3.733 ± 8.196↑	0.008	3.251_LMS_
2		Bifidobacteriales	*Bifidobacterium*	/	0.049 ± 0.083	0.018 ± 0.043↓	0.013 ± 0.033↓	0.066 ± 0.125↑	0.062 ± 0.152↑	3.702 ± 8.160↑	0.008	3.250_LMS_
3				*Bifidobacterium_pseudocatenulatum*	0.009 ± 0.022	0.000 ± 0.000↓	0.000 ± 0.000↓	0.004 ± 0.011↓	0.058 ± 0.141↑	0.010 ± 0.022↑	0.003	3.249_LMS_
4	Bacteroidetes	Bacteroidales	*/*	/	80.96 ± 73.19	20.24 ± 15.21↓	5.199 ± 4.148↓	3.707 ± 2.010↓	12.12 ± 13.62↓	8.313 ± 1.567↓	0.001	4.594_CK_
5			*Bacteroides*	/	43.96 ± 21.44	117.4 ± 134.2↑	110.4 ± 30.03↑	47.40 ± 27.08↑	47.23 ± 15.77↑	20.68 ± 7.947↓	0.003	4.675_L-GLPP_
6				*Bacteroides_vulgatus*	0.221 ± 0.163	1.665 ± 2.125↑	1.200 ± 1.786↑	0.252 ± 0.196↑	0.306 ± 0.282↑	0.084 ± 0.108↓	0.007	2.905_CTX_
7				*Bacteroides_intestinalis*	0.173 ± 0.111	0.682 ± 1.039↑	0.819 ± 0.957↑	0.208 ± 0.186↑	0.208 ± 0.278↑	0.049 ± 0.026↓	0.004	2.654_L-GLPP_
8			*Odoribacter*	/	19.68 ± 7.980	44.75 ± 24.35↑	84.08 ± 87.20↑	40.72 ± 59.84↑	32.26 ± 38.71↑	13.89 ± 6.345↓	0.032	4.563_L-GLPP_
9			*/*	/	54.30 ± 20.17	69.62 ± 41.35↑	115.3 ± 68.06↑	74.89 ± 26.18↑	52.24 ± 17.93↓	55.64 ± 25.46↑	0.025	3.831_L-GLPP_
10			*Alistipes*	/	46.85 ± 18.90	58.75 ± 34.17↑	88.43 ± 64.94↑	61.19 ± 21.01↑	42.11 ± 13.61↓	45.07 ± 23.10↓	0.004	4.316_L-GLPP_
11			*Rikenella*	/	4.402 ± 1.993	9.925 ± 7.740↑	11.81 ± 7.099↑	9.194 ± 5.196↑	7.144 ± 6.335↑	6.506 ± 3.994↑	0.005	3.436_L-GLPP_
12			*Parabacteroides*	/	3.401 ± 0.558	4.066 ± 2.773↑	14.85 ± 15.48↑	4.296 ± 2.262↑	2.095 ± 1.199↓	2.210 ± 0.818↓	0.002	3.755_L-GLPP_
13				Parabacteroides_distasonis	2.175 ± 0.353	2.578 ± 1.790↑	4.229 ± 2.568↑	1.958 ± 1.576↓	1.138 ± 0.678↓	1.267 ± 0.455↓	0.034	3.166_L-GLPP_
14	Firmicutes	Bacillales	*Staphylococcus*	/	0.031 ± 0.043	0.093 ± 0.140↑	0.044 ± 0.032↑	0.000 ± 0.000↓	0.000 ± 0.000↓	0.027 ± 0.034↓	0.011	1.880_CTX_
15		/	*/*	/	323.3 ± 92.67	285.8 ± 92.92↓	304.8 ± 116.3↓	409.3 ± 61.33↑	351.3 ± 195.5↑	346.2 ± 116.6↑	0.039	1.844_LMS_
16		Clostridiales	*Roseburia*	/	9.628 ± 6.485	8.326 ± 5.419↓	3.707 ± 3.101↓	6.683 ± 3.480↓	14.53 ± 6.759↑	7.427 ± 3.902↓	0.035	3.754_H-GLPP_
17			*Lachnospiraceae*	*[Clostridium]_colinum*	0.775 ± 1.214	0.301 ± 0.244↓	0.186 ± 0.126↓	0.664 ± 0.521↓	0.983 ± 1.890↑	1.098 ± 0.669↑	0.029	2.691_H-GLPP_
18				*Lachnospiraceae_bacterium_COE1*	1.196 ± 0.588	1.222 ± 0.753↑	0.319 ± 0.244↓	1.404 ± 1.376↑	2.941 ± 2.599↑	0.607 ± 0.417↓	0.023	3.135_H-GLPP_
19				*Lachnospiraceae_bacterium_A4*	0.673 ± 0.552	1.603 ± 3.283↑	0.062 ± 0.036↓	0.261 ± 0.196↓	0.447 ± 0.374↓	0.190 ± 0.117↓	0.022	2.896_H-GLPP_
20			*/*	/	0.005 ± 0.011	0.000 ± 0.000↓	0.009 ± 0.022↑	0.018 ± 0.024↑	0.186 ± 0.404↑	0.003 ± 0.011↓	0.042	2.076_H-GLPP_
21			*Romboutsia*	/	0.004 ± 0.011	0.000 ± 0.000↓	0.009 ± 0.022↑	0.018 ± 0.027↑	0.173 ± 0.372↑	0.002 ± 0.011↓	0.031	2.050_H-GLPP_
22			*Negativibacillus*	/	0.695 ± 0.314	0.686 ± 0.486↓	0.128 ± 0.078↓	0.523 ± 0.338↓	0.700 ± 0.452	0.602 ± 0.128↓	0.023	2.477_CK_
23		Erysipelotrichales	*Ileibacterium*	*Ileibacterium_valens*	0.000 ± 0.000	0.000 ± 0.000	0.186 ± 0.430↑	0.018 ± 0.027↑	0.004 ± 0.011↑	0.000 ± 0.000	0.034	2.084_M-GLPP_
24	Proteobacteria	/	*/*	/	0.766 ± 0.163	3.211 ± 2.642↑	3.193 ± 3.920↑	0.735 ± 0.401↓	0.740 ± 0.244↓	0.580 ± 0.574↓	0.007	2.692_CTX_
25		Desulfovibrionales	*/*	/	5.722 ± 5.243	2.059 ± 1.445↓	3.432 ± 3.964↓	2.321 ± 1.032↓	2.325 ± 1.213↓	4.349 ± 5.099↓	0.004	3.264_CK_
26		Enterobacteriales	*/*	/	0.208 ± 0.078	1.085 ± 2.204↑	0.483 ± 0.853↑	0.089 ± 0.022↓	0.207 ± 0.215↓	0.062 ± 0.057↓	0.025	2.706_CK_
27			*Enterobacteriaceae*	/	0.186 ± 0.067	1.032 ± 2.203↑	0.469 ± 0.858↑	0.066 ± 0.028↓	0.164 ± 0.202↓	0.035 ± 0.036↓	0.017	2.692_CK_
28		Pasteurellales	*/*	/	0.027 ± 0.044	0.017 ± 0.030↓	1.754 ± 3.304↑	0.036 ± 0.029↑	0.044 ± 0.108↑	0.035 ± 0.054↑	0.014	2.845_L-GLPP_
29			*Rodentibacter*	/	0.022 ± 0.043	0.018 ± 0.032↓	1.758 ± 3.314↑	0.035 ± 0.032↑	0.000 ± 0.000↓	0.035 ± 0.057↑	0.003	2.861_L-GLPP_
30		Rhodospirillales	*/*	/	0.611 ± 0.670	0.226 ± 0.292↓	2.108 ± 3.065↑	0.195 ± 0.203↓	0.035 ± 0.064↓	0.505 ± 0.418↓	0.007	3.037_CK_
31		Sphingomonadales	*Sphingomonas*	/	0.058 ± 0.059	0.013 ± 0.022↓	0.053 ± 0.082↓	0.089 ± 0.067↑	0.093 ± 0.115↑	0.000 ± 0.000↓	0.020	1.823_M-GLPP_
32		Gammaproteobacteria	*/*	/	0.478 ± 0.091	2.037 ± 1.313↑	0.930 ± 0.795↑	0.598 ± 0.385↑	0.452 ± 0.099↓	0.474 ± 0.607↓	0.014	2.908 _CTX_
33			*Parasutterella*	/	0.465 ± 0.090	2.006 ± 1.300↑	0.899 ± 0.794↑	0.571 ± 0.391↑	0.416 ± 0.105↓	0.452 ± 0.606↓	0.012	2.918 _CTX_
34	Tenericutes	Anaeroplasmatales	*Anaeroplasma*	/	1.577 ± 1.389	16.27 ± 22.39↑	6.072 ± 12.46↑	0.514 ± 0.464↓	1.107 ± 0.710↓	0.678 ± 0.769↓	0.046	3.884 _CTX_

Compared with the CK group, red upward arrows indicate an increase in Relative Abundance, and green downward arrows indicate a decrease in Relative Abundance.

**Table 2:** Putatively annotated differential metabolite features in cecal contents of BALB/c mice after GLPP and LMS intervention.

KEGG ID	Class	Metabolite	MSI Level	Modes	VIP	FC	Trends	Group
C03406	Carboxylic acids and derivatives	Argininosuccinic acid	2	Negative	1.26	6.88	up	CK vs. H-GLPP
C00158	Carboxylic acids and derivatives	Citric acid	2	Negative	1.09	2.23	up	CK vs. H-GLPP
C00624	Carboxylic acids and derivatives	N-Acetylglutamic acid	2	Negative	1.03	2.32	up	CK vs. H-GLPP
C00383	Carboxylic acids and derivatives	Malonic acid	2	Negative	1	4.85	up	CK vs. H-GLPP
-	Carboxylic acids and derivatives	N-Acetylalanine	2	Positive	1.14	2.6	up	CK vs. H-GLPP
C05824	Carboxylic acids and derivatives	S-Sulfocysteine	2	Positive	1.06	0.45	down	CK vs. H-GLPP
-	Fatty Acyls	2-Hydroxyisocaproic acid	2	Negative	1.01	2.28	up	CK vs. H-GLPP
C00696	Fatty Acyls	Prostaglandin D2	2	Negative	1.01	2.04	up	CK vs. H-GLPP
C00219	Fatty Acyls	Arachidonic acid	2	Negative	1.01	3.05	up	CK vs. H-GLPP
C02990	Fatty Acyls	Palmitoylcarnitine	2	Positive	1.18	0	down	CK vs. H-GLPP
C01909	Fatty Acyls	Dethiobiotin	2	Positive	1.09	0.47	down	CK vs. H-GLPP
C05951	Fatty Acyls	Leukotriene D4	2	Positive	1.05	0.2	down	CK vs. H-GLPP
C00093	Glycerophospholipids	Glycerol 3-phosphate	2	Negative	1.21	5.61	up	CK vs. H-GLPP
C01013	Hydroxy acids and derivatives	3-Hydroxypropanoic acid	2	Negative	1.04	2.1	up	CK vs. H-GLPP
C00955	Indoles and derivatives	Tryptophol	2	Negative	1.08	2.87	up	CK vs. H-GLPP
C00092	Organooxygen compounds	Glucose 6-phosphate	2	Negative	1.25	6.37	up	CK vs. H-GLPP
C00117	Organooxygen compounds	Ribulose 5-phosphate	2	Negative	1.23	4.9	up	CK vs. H-GLPP
C00231	Organooxygen compounds	Xylulose 5-phosphate	2	Negative	1.19	5.24	up	CK vs. H-GLPP
C00352	Organooxygen compounds	Glucosamine 6-phosphate	2	Negative	1.16	2.91	up	CK vs. H-GLPP
C00493	Organooxygen compounds	Shikimic acid	2	Negative	1.14	0.48	down	CK vs. H-GLPP
C00388	Organonitrogen compounds	Histamine	2	Positive	1.2	0.48	down	CK vs. H-GLPP
C03672	Phenylpropanoic acids	Hydroxyphenyllactic acid	2	Negative	1.03	2.4	up	CK vs. H-GLPP
-	Phenylpropanoic acids	3-(3-Hydroxyphenyl)-3-hydroxypropanoic acid	2	Negative	1.02	0.4	down	CK vs. H-GLPP
C00301	Purine nucleosides	ADP-ribose	2	Negative	1.16	2.89	up	CK vs. H-GLPP
C00029	Purine nucleosides	UDP-glucose	2	Negative	1.08	3.77	up	CK vs. H-GLPP
C00020	Purine nucleotides	Adenosine 5′-monophosphate	2	Negative	1.03	2.58	up	CK vs. H-GLPP
C00362	Purine nucleotides	2′-Deoxyguanosine 5′-monophosphate	2	Negative	1.01	2.96	up	CK vs. H-GLPP
C00942	Purine nucleotides	Guanosine 3′,5′-cyclic monophosphate	2	Positive	1.04	2.15	up	CK vs. H-GLPP
C05843	Pyridines and derivatives	1,4-Dihydro-1-methyl-4-oxo-3-pyridinecarboxamide	2	Positive	1.01	2.48	up	CK vs. H-GLPP
C01367	Ribonucleoside 3′-phosphates	3′-Adenylic acid	2	Positive	1.11	2.82	up	CK vs. H-GLPP
C03406	Carboxylic acids and derivatives	Argininosuccinic acid	2	Negative	1.13	2.47	up	CTX vs. H-GLPP
-	Carboxylic acids and derivatives	gamma-Glutamylleucine	2	Negative	1.01	2.03	up	CTX vs. H-GLPP
-	Carboxylic acids and derivatives	2-Aminooctanoic acid	2	Positive	1.01	2.07	up	CTX vs. H-GLPP
-	Cinnamic acids and derivatives	4-Methoxycinnamic acid	2	Negative	1.14	0.45	down	CTX vs. H-GLPP
C00584	Fatty Acyls	Prostaglandin E2	2	Negative	1.28	2.81	up	CTX vs. H-GLPP
C02990	Fatty Acyls	Palmitoylcarnitine	2	Positive	1.09	0.19	down	CTX vs. H-GLPP
C05951	Fatty Acyls	Leukotriene D4	2	Positive	1.26	0.09	down	CTX vs. H-GLPP
-	Glycerophospholipids	LysoPC 16:1	2	Positive	1.17	2.17	up	CTX vs. H-GLPP
-	Glycerophospholipids	LysoPC 14:0	2	Positive	1.11	2.09	up	CTX vs. H-GLPP
C00977	Indoles and derivatives	Tryptophanamide	2	Positive	1.08	2.07	up	CTX vs. H-GLPP
-	Organooxygen compounds	4-Hydroxycyclohexylcarboxylic acid	2	Negative	1.29	0.27	down	CTX vs. H-GLPP
C00493	Organooxygen compounds	Shikimic acid	2	Negative	1.13	2.54	up	CTX vs. H-GLPP
C00117	Organooxygen compounds	Ribulose 5-phosphate	2	Negative	1.1	2.03	up	CTX vs. H-GLPP
C00092	Organooxygen compounds	Glucose 6-phosphate	2	Negative	1.08	2.24	up	CTX vs. H-GLPP
C02406	Organooxygen compounds	N’-Formylkynurenine	2	Negative	1.07	2.11	up	CTX vs. H-GLPP
-	Phenylpropanoic acids	3-(3-Hydroxyphenyl)-3-hydroxypropanoic acid	2	Negative	1.16	0.44	down	CTX vs. H-GLPP
C05512	Purine nucleosides	2′-Deoxyinosine	2	Positive	1.14	0.47	down	CTX vs. H-GLPP
C00559	Purine nucleosides	Deoxyadenosine	2	Positive	1.13	0.5	down	CTX vs. H-GLPP
C02140	Steroids and steroid derivatives	Corticosterone	2	Negative	1.15	0.48	down	CTX vs. H-GLPP
C01586	Benzene and substituted derivatives	Hippuric acid	2	Positive	1.1	6.84	up	CTX vs. H-GLPP
C00331	-	Indole-3-pyruvic acid	2	Negative	1.11	2.14	up	CTX vs. LMS
C00003	(5′->5′)-dinucleotides	Nicotinic acid adenine dinucleotide	2	Positive	1.08	2.55	up	CTX vs. LMS
C00170	5′-deoxyribonucleosides	5′-Methylthioadenosine	2	Positive	1.09	4.24	up	CTX vs. LMS
-	5′-deoxyribonucleosides	S-Adenosylmethionine	2	Positive	1.09	2.9	up	CTX vs. LMS
C00628	Benzene and substituted derivatives	2,5-Dihydroxybenzoic acid	2	Negative	1.02	3.33	up	CTX vs. LMS
-	Benzene and substituted derivatives	2,4-Dihydroxybenzoic acid	2	Negative	1	1234.03	up	CTX vs. LMS
C00230	Benzene and substituted derivatives	Protocatechuic acid	2	Negative	1.03	24.38	up	CTX vs. LMS
C02946	Carboxylic acids and derivatives	4-Acetamidobutyric acid	2	Positive	1.13	2.47	up	CTX vs. LMS
C00300	Carboxylic acids and derivatives	Creatine	2	Negative	1.12	0.27	down	CTX vs. LMS
-	Carboxylic acids and derivatives	N-Acetylalanine	2	Positive	1.11	0.27	down	CTX vs. LMS
C05608	Cinnamaldehydes	*p*-Coumaraldehyde	2	Positive	1.01	3.45	up	CTX vs. LMS
C09276	Coumarins and derivatives	Marmesin	2	Negative	1.09	3.88	up	CTX vs. LMS
-	Fatty Acyls	5-HEPE	2	Negative	1.05	2.42	up	CTX vs. LMS
C02678	Fatty Acyls	Dodecanedioic acid	2	Negative	1.14	2.38	up	CTX vs. LMS
C14827	Fatty Acyls	9-HpODE	2	Negative	1.05	2.97	up	CTX vs. LMS
C02990	Fatty Acyls	Palmitoylcarnitine	2	Positive	1.13	0.04	down	CTX vs. LMS
C02571	Fatty Acyls	Acetylcarnitine	2	Positive	1.07	0.29	down	CTX vs. LMS
C05951	Fatty Acyls	Leukotriene D4	2	Positive	1.11	0.1	down	CTX vs. LMS
C00016	Flavin nucleotides	Flavin adenine dinucleotide	2	Negative	1.07	2.09	up	CTX vs. LMS
C00416	Glycerophospholipids	LysoPA 16:0	2	Negative	1.33	0.46	down	CTX vs. LMS
-	Glycerophospholipids	LysoPE 18:0	2	Negative	1.04	0.38	down	CTX vs. LMS
-	Glycerophospholipids	LysoPC 20:2	2	Positive	1.35	0.38	down	CTX vs. LMS
-	Glycerophospholipids	LysoPC 14:0	2	Positive	1.33	0.46	down	CTX vs. LMS
-	Glycerophospholipids	LysoPC 18:1	2	Positive	1.31	0.49	down	CTX vs. LMS
-	Glycerophospholipids	LysoPC 20:1	2	Positive	1.31	0.37	down	CTX vs. LMS
-	Glycerophospholipids	LysoPC 16:0	2	Positive	1.3	0.45	down	CTX vs. LMS
-	Glycerophospholipids	LysoPC 16:1	2	Positive	1.3	0.32	down	CTX vs. LMS
-	Glycerophospholipids	LysoPC 18:3	2	Positive	1.28	0.44	down	CTX vs. LMS
-	Glycerophospholipids	LysoPC 18:0	2	Positive	1.27	0.31	down	CTX vs. LMS
-	Glycerophospholipids	PAF C-16	2	Positive	1.26	0.31	down	CTX vs. LMS
-	Glycerophospholipids	LysoPC 15:0	2	Positive	1.24	0.36	down	CTX vs. LMS
-	Glycerophospholipids	LysoPC 17:0	2	Positive	1.24	0.38	down	CTX vs. LMS
C00670	Glycerophospholipids	Glycerophosphatidylcholine	2	Positive	1.04	0.22	down	CTX vs. LMS
C00186	Hydroxy acids and derivatives	Lactic acid	2	Negative	1.16	0.5	down	CTX vs. LMS
C07480	Imidazopyrimidines	Theobromine	2	Positive	1.12	0.49	down	CTX vs. LMS
C05635	Indoles and derivatives	5-Hydroxyindole-3-acetic acid	2	Positive	1.03	5.67	up	CTX vs. LMS
-	Indoles and derivatives	3-Indolepropionic acid	2	Negative	1.09	2.19	up	CTX vs. LMS
C00955	Indoles and derivatives	Tryptophol	2	Negative	1.07	0.31	down	CTX vs. LMS
-	Lactones	Erythrono-1,4-lactone	2	Negative	1.12	2.38	up	CTX vs. LMS
C00315	Organonitrogen compounds	Spermidine	2	Positive	1.09	5.34	up	CTX vs. LMS
C02640	Organonitrogen compounds	3-Methyl-1-butylamine	2	Positive	1.07	2553.39	up	CTX vs. LMS
C04256	Organooxygen compounds	N-Acetylglucosamine 1-phosphate	2	Negative	1.13	0.45	down	CTX vs. LMS
C19910	Organooxygen compounds	N-Acetylneuraminic acid	2	Positive	1.17	0.47	down	CTX vs. LMS
C11457	Phenylpropanoic acids	3-(3-Hydroxyphenyl)propionic acid	2	Negative	1.07	8.95	up	CTX vs. LMS
C05607	Phenylpropanoic acids	3-Phenyllactic acid	2	Negative	1.06	4.7	up	CTX vs. LMS
C05629	Phenylpropanoic acids	Hydrocinnamic acid	2	Negative	1	4.62	up	CTX vs. LMS
C00899	Prenol lipids	11-cis-Retinol	2	Positive	1.03	5.98	up	CTX vs. LMS
C00301	Purine nucleosides	ADP-ribose	2	Negative	1.1	2.13	up	CTX vs. LMS
-	Purine nucleosides	N6-Succinyl adenosine	2	Positive	1.25	0.45	down	CTX vs. LMS
C00314	Pyridines and derivatives	Pyridoxine	2	Positive	1.1	3.51	up	CTX vs. LMS
C00153	Pyridines and derivatives	Nicotinamide	2	Positive	1.07	2.3	up	CTX vs. LMS
-	Pyridines and derivatives	6-Methylnicotinamide	2	Positive	1.16	2.13	up	CTX vs. LMS
C05942	Pyrroles	Pyrrole-2-carboxylic acid	2	Negative	1.05	2.92	up	CTX vs. LMS
C02470	Quinolines and derivatives	Xanthurenic acid	2	Positive	1.02	9.63	up	CTX vs. LMS
C05465	Steroids and steroid derivatives	Taurochenodesoxycholic acid	2	Negative	1.02	0.35	down	CTX vs. LMS
C02538	Steroids and steroid derivatives	Estrone 3-sulfate	2	Negative	1.01	5.23	up	CTX vs. LMS

## Data Availability

The original contributions presented in this study are included in the article/Supplementary Material. Further inquiries can be directed to the corresponding authors.

## Supplementary Materials

The following supporting information can be downloaded at: https://www.mdpi.com/article/10.3390/foods15132370/s1, Figure S1: KEGG enrichment of DEGs in CTX vs. H-GLPP; Figure S2: KEGG enrichment of DEGs in CTX vs. LMS; Figure S3A: Hierarchical clustering heatmap of differential metabolites; Figure S3B: Metabolite–metabolite correlation heatmap; Figure S4: Hierarchical clustering heatmap of gene expression profiles; Figure S5A–C: Full Mantel test microbiota–metabolite association maps at kingdom, phylum, and order levels; Figure S5D: Full metabolite-DEG Pearson correlation heatmap; Table S1: Median and interquartile range of differential gut microorganisms; Table S2: Metabolite annotation confidence for all reported differential metabolites.

## Abbreviations

The following abbreviations are used in this manuscript:

Arg-Pro	arginine–proline
ANOVA	analysis of variance
AhR	aryl hydrocarbon receptor
CCK-8	Cell Counting Kit-8
CTX	cyclophosphamide
DEGs	differentially expressed genes
DTH	delayed-type hypersensitivity
FDR	false discovery rate
FPKM	fragments per kilobase of transcript per million mapped reads
GLPP	*Ganoderma lucidum* polysaccharide peptide
H&E	hematoxylin and eosin
IFN-γ	interferon-γ
IgA	immunoglobulin A
IL-2	interleukin-2
LEfSe	linear discriminant analysis effect size
LMS	levamisole hydrochloride
LTD4	leukotriene D4
MAPK	mitogen-activated protein kinase
NF-κB	nuclear factor kappa B
OTUs	operational taxonomic units
PCA	principal component analysis
PCoA	principal coordinate analysis
PLS-DA	partial least squares–discriminant analysis
PRRs	pattern recognition receptors
SDS	sodium dodecyl sulfate
TNF-α	tumor necrosis factor-α
UPLC-QTrap-MS/MS	ultra-performance liquid chromatography–tandem mass spectrometry
VIP	variable importance in projection
