# Supplementary material for: Integrated Foodomics Reveals Gut Microbiota–Metabolite–Gene Interactions Associated with the Immunoprotective Effects of Ganoderma lucidum Polysaccharide Peptide
Source: Foods. 2026 Jul 3;15(13):2370. doi: 10.3390/foods15132370 (PMC13361114; doi:10.3390/foods15132370)
Supplement: Supplementary file 1 [file foods-15-02370-s001.zip › Supplementary materials legends.pdf]

Supplementary Figure legends

Figure S1. KEGG pathway enrichment analysis of DEGs in CTX vs. H-GLPP comparison.

Figure S2. KEGG pathway enrichment analysis of DEGs in CTX vs. LMS comparison.

Figure S3A. Hierarchical clustering heatmap of differential metabolites in cecal contents. Each row represents a differential metabolite, and each column represents an individual mouse sample. The color scale indicates Z-score-normalized metabolite abundance (red: high abundance; blue: low abundance). Samples are grouped by experimental condition (CK, CTX, L-GLPP, M-GLPP, H-GLPP, LMS).

Figure S3B. Metabolite-metabolite correlation heatmap based on Pearson analysis of 56 differential metabolites between CTX and LMS groups. Red: positive correlation; blue: negative correlation.

Figure S4A. Hierarchical clustering heatmap of cecal gene expression profiles. Rows represent genes, columns represent samples; Z-score normalized expression is shown (red: high; blue: low). Samples are organized by experimental group (CK, CTX, L-GLPP, M-GLPP, H-GLPP, LMS).

Figure S5A. Mantel-test associations between gut microbiota and host metabolites at the Kingdom level. Line color and width indicate correlation strength and significance as defined in the figure.

Figure S5B. Mantel-test associations between gut microbiota and host metabolites at the Phylum level. Line color and width indicate correlation strength and significance as defined in the figure.

Figure S5C. Mantel-test associations between gut microbiota and host metabolites at the Order level. Line color and width indicate correlation strength and significance as defined in the figure.

Figure S5D. Full Pearson correlation heatmap between differential metabolites and DEGs. The matrix shows correlation coefficients between 92 differential metabolites and 78 DEGs. Line color and width indicate correlation strength and significance as defined in the figure.

Supplementary Table legends

Table S1. Median and interquartile range (IQR) of selected bacterial taxa showing skewed distribution (SD > mean) across experimental groups. Data are normalized to $\times 10^3$ for direct comparison with Table 1.

Table S2. Metabolite annotation confidence for all reported differential metabolites.
