# Supplementary material for: Integrated Foodomics Reveals Gut Microbiota–Metabolite–Gene Interactions Associated with the Immunoprotective Effects of Ganoderma lucidum Polysaccharide Peptide
Source: Foods. 2026 Jul 3;15(13):2370. doi: 10.3390/foods15132370 (PMC13361114; doi:10.3390/foods15132370)
Supplement: Supplementary file 1 [file foods-15-02370-s001.zip › Table S1.pdf]

Table S1. Median and interquartile range (IQR) of selected bacterial taxa showing skewed distribution (SD > mean) in each group

Taxon	Group	Median (×10³)	Q1 – Q3 (×10³)	IQR
Bifidobacterium	CK	0	0 – 0	0
	CTX	0	0 – 0	0
	L-GLPP	0	0 – 0	0
	M-GLPP	0	0 – 0	0
	H-GLPP	0	0 – 0	0
	LMS	0	0 – 0	0
Bacteroides	CK	43.96	38.2 – 49.7	11.5
	CTX	117.4	45.3 – 189.6	144.3
	L-GLPP	110.4	89.2 – 131.6	42.4
	M-GLPP	47.4	42.1 – 52.7	10.6
	H-GLPP	47.23	41.0 – 53.5	12.5
	LMS	20.68	15.2 – 26.1	10.9
Odoribacter	CK	19.68	14.5 – 24.9	10.4
	CTX	44.75	28.3 – 61.2	32.9
	L-GLPP	84.08	42.1 – 126.1	84
	M-GLPP	40.72	28.9 – 52.5	23.6
	H-GLPP	32.26	23.8 – 40.7	16.9
	LMS	13.89	8.5 – 19.3	10.8
Alistipes	CK	46.85	35.2 – 58.5	23.3
	CTX	58.75	32.1 – 85.4	53.3
	L-GLPP	88.43	44.6 – 132.3	87.7
	M-GLPP	61.19	47.5 – 74.9	27.4
	H-GLPP	42.11	33.8 – 50.4	16.6
	LMS	45.07	31.2 – 58.9	27.7
Anaeroplasma	CK	1.577	0.8 – 2.3	1.5
	CTX	16.27	5.2 – 27.3	22.1
	L-GLPP	6.072	1.1 – 11.0	9.9
	M-GLPP	0.514	0.2 – 0.8	0.6
	H-GLPP	1.107	0.5 – 1.7	1.2
	LMS	0.678	0.3 – 1.1	0.8
Parabacteroides	CK	3.401	2.9 – 3.9	1
	CTX	4.066	2.8 – 5.3	2.5
	L-GLPP	14.85	6.2 – 23.5	17.3
	M-GLPP	4.296	3.2 – 5.4	2.2
	H-GLPP	2.095	1.5 – 2.7	1.2
	LMS	2.21	1.7 – 2.7	1
Parabacteroides distasonis	CK	2.175	1.9 – 2.4	0.5
	CTX	2.578	1.5 – 3.7	2.2
	L-GLPP	4.229	2.8 – 5.7	2.9
	M-GLPP	1.958	1.3 – 2.6	1.3
	H-GLPP	1.138	0.8 – 1.5	0.7
	LMS	1.267	1.0 – 1.5	0.5
Rikenella	CK	4.402	3.2 – 5.6	2.4
	CTX	9.925	5.5 – 14.4	8.9
	L-GLPP	11.81	7.2 – 16.4	9.2

	M-GLPP	9.194	5.8 – 12.6	6.8
	H-GLPP	7.144	3.5 – 10.8	7.3
	LMS	6.506	4.1 – 8.9	4.8
Lachnospiraceae bacterium COE1	CK	1.196	0.8 – 1.6	0.8
	CTX	1.222	0.7 – 1.7	1
	L-GLPP	0.319	0.2 – 0.4	0.2
	M-GLPP	1.404	0.8 – 2.0	1.2
	H-GLPP	2.941	1.5 – 4.4	2.9
	LMS	0.607	0.4 – 0.8	0.4

Data are presented as median and interquartile range (Q1–Q3) based on raw OTU counts normalized to $\times 10^3$ for direct comparison with Table 1. For taxa not listed, unambiguous OTU assignment was not feasible.
