# Supplementary material for: Integrated Foodomics Reveals Gut Microbiota–Metabolite–Gene Interactions Associated with the Immunoprotective Effects of Ganoderma lucidum Polysaccharide Peptide
Source: Foods. 2026 Jul 3;15(13):2370. doi: 10.3390/foods15132370 (PMC13361114; doi:10.3390/foods15132370)
Supplement: Supplementary file 1 [file foods-15-02370-s001.zip › Table S2.pdf]

Table S2. Metabolite annotation confidence for all reported differential metabolites.

Metabolite	HMDB ID	KEGG ID	Class	Annotation Basis	MSI Level	Modes	VIP	FC	Trends	Group
Argininosuccinic acid	HMDB0000052	C03406	Carboxylic acids and derivatives	MWDB + MS/MS	2	Negative	1.26	6.88	up	CK vs. H-GLPP
Citric acid	HMDB0000094	C00158	Carboxylic acids and derivatives	MWDB + MS/MS	2	Negative	1.09	2.23	up	CK vs. H-GLPP
N-Acetylglutamic acid	HMDB0001138	C00624	Carboxylic acids and derivatives	MWDB + MS/MS	2	Negative	1.03	2.32	up	CK vs. H-GLPP
Malonic acid	HMDB0000691	C00383	Carboxylic acids and derivatives	MWDB + MS/MS	2	Negative	1	4.85	up	CK vs. H-GLPP
N-Acetylalanine	HMDB0000766	-	Carboxylic acids and derivatives	MWDB + MS/MS	2	Positive	1.14	2.6	up	CK vs. H-GLPP
S-Sulfocysteine	HMDB0000733	C05824	Carboxylic acids and derivatives	MWDB + MS/MS	2	Positive	1.06	0.45	down	CK vs. H-GLPP
2-Hydroxyisocaproic acid	HMDB0000746	-	Fatty Acyls	MWDB + MS/MS	2	Negative	1.01	2.28	up	CK vs. H-GLPP
Prostaglandin D2	HMDB0001403	C00696	Fatty Acyls	MWDB + MS/MS	2	Negative	1.01	2.04	up	CK vs. H-GLPP
Arachidonic acid	HMDB0001043	C00219	Fatty Acyls	MWDB + MS/MS	2	Negative	1.01	3.05	up	CK vs. H-GLPP
Palmitoylcarnitine	HMDB0000222	C02990	Fatty Acyls	MWDB + MS/MS	2	Positive	1.18	0	down	CK vs. H-GLPP
Dethiobiotin	HMDB0000646	C01909	Fatty Acyls	MWDB + MS/MS	2	Positive	1.09	0.47	down	CK vs. H-GLPP
Leukotriene D4	HMDB0003080	C05951	Fatty Acyls	MWDB + MS/MS	2	Positive	1.05	0.2	down	CK vs. H-GLPP
Glycerol 3-phosphate	HMDB0000126	C00093	Glycerophospholipids	MWDB + MS/MS	2	Negative	1.21	5.61	up	CK vs. H-GLPP
3-Hydroxypropanoic acid	HMDB0000700	C01013	Hydroxy acids and derivatives	MWDB + MS/MS	2	Negative	1.04	2.1	up	CK vs. H-GLPP
Tryptophol	HMDB0003447	C00955	Indoles and derivatives	MWDB + MS/MS	2	Negative	1.08	2.87	up	CK vs. H-GLPP
Glucose 6-phosphate	HMDB0001401	C00092	Organooxygen compounds	MWDB + MS/MS	2	Negative	1.25	6.37	up	CK vs. H-GLPP
Ribulose 5-phosphate	HMDB0000618	C00117	Organooxygen compounds	MWDB + MS/MS	2	Negative	1.23	4.9	up	CK vs. H-GLPP
Xylulose 5-phosphate	HMDB0000868	C00231	Organooxygen compounds	MWDB + MS/MS	2	Negative	1.19	5.24	up	CK vs. H-GLPP
Glucosamine 6-phosphate	HMDB0001254	C00352	Organooxygen compounds	MWDB + MS/MS	2	Negative	1.16	2.91	up	CK vs. H-GLPP
Shikimic acid	HMDB0003070	C00493	Organooxygen compounds	MWDB + MS/MS	2	Negative	1.14	0.48	down	CK vs. H-GLPP
Histamine	HMDB0000870	C00388	Organonitrogen compounds	MWDB + MS/MS	2	Positive	1.2	0.48	down	CK vs. H-GLPP
Hydroxyphenyllactic acid	HMDB0000755	C03672	Phenylpropanoic acids	MWDB + MS/MS	2	Negative	1.03	2.4	up	CK vs. H-GLPP
3-(3-Hydroxyphenyl)-3-hydroxypropanoic acid	-	-	Phenylpropanoic acids	MWDB + MS/MS	2	Negative	1.02	0.4	down	CK vs. H-GLPP
ADP-ribose	HMDB0001178	C00301	Purine nucleosides	MWDB + MS/MS	2	Negative	1.16	2.89	up	CK vs. H-GLPP
UDP-glucose	HMDB0000286	C00029	Purine nucleosides	MWDB + MS/MS	2	Negative	1.08	3.77	up	CK vs. H-GLPP
Adenosine 5'-monophosphate	HMDB0000045	C00020	Purine nucleotides	MWDB + MS/MS	2	Negative	1.03	2.58	up	CK vs. H-GLPP
2'-Deoxyguanosine 5'-monophosphate	HMDB0001044	C00362	Purine nucleotides	MWDB + MS/MS	2	Negative	1.01	2.96	up	CK vs. H-GLPP
Guanosine 3',5'-cyclic monophosphate	HMDB0001314	C00942	Purine nucleotides	MWDB + MS/MS	2	Positive	1.04	2.15	up	CK vs. H-GLPP
1,4-Dihydro-1-methyl-4-oxo-3-pyridinecarboxamide	-	C05843	Pyridines and derivatives	MWDB + MS/MS	2	Positive	1.01	2.48	up	CK vs. H-GLPP
3'-Adenylic acid	-	C01367	Ribonucleoside 3'-phosphates	MWDB + MS/MS	2	Positive	1.11	2.82	up	CK vs. H-GLPP
Argininosuccinic acid	HMDB0000052	C03406	Carboxylic acids and derivatives	MWDB + MS/MS	2	Negative	1.13	2.47	up	CTX vs. H-GLPP
gamma-Glutamylleucine	HMDB0011171	-	Carboxylic acids and derivatives	MWDB + MS/MS	2	Negative	1.01	2.03	up	CTX vs. H-GLPP
2-Aminooctanoic acid	HMDB0000991	-	Carboxylic acids and derivatives	MWDB + MS/MS	2	Positive	1.01	2.07	up	CTX vs. H-GLPP
4-Methoxycinnamic acid	HMDB0246971	-	Cinnamic acids and derivatives	MWDB + MS/MS	2	Negative	1.14	0.45	down	CTX vs. H-GLPP
Prostaglandin E2	HMDB0001220	C00584	Fatty Acyls	MWDB + MS/MS	2	Negative	1.28	2.81	up	CTX vs. H-GLPP
Palmitoylcarnitine	HMDB0000222	C02990	Fatty Acyls	MWDB + MS/MS	2	Positive	1.09	0.19	down	CTX vs. H-GLPP
Leukotriene D4	HMDB0003080	C05951	Fatty Acyls	MWDB + MS/MS	2	Positive	1.26	0.09	down	CTX vs. H-GLPP
LysoPC 16:1	-	-	Glycerophospholipids	MWDB + MS/MS	2	Positive	1.17	2.17	up	CTX vs. H-GLPP
LysoPC 14:0	-	-	Glycerophospholipids	MWDB + MS/MS	2	Positive	1.11	2.09	up	CTX vs. H-GLPP
Shikimic acid	HMDB0013318	C00977	Indoles and derivatives	MWDB + MS/MS	2	Positive	1.08	2.07	up	CTX vs. H-GLPP
4-Hydroxycyclohexylcarboxylic acid	-	-	Organooxygen compounds	MWDB + MS/MS	2	Negative	1.29	0.27	down	CTX vs. H-GLPP
Shikimic acid	HMDB0003070	C00493	Organooxygen compounds	MWDB + MS/MS	2	Negative	1.13	2.54	up	CTX vs. H-GLPP
Ribulose 5-phosphate	HMDB0000618	C00117	Organooxygen compounds	MWDB + MS/MS	2	Negative	1.1	2.03	up	CTX vs. H-GLPP

Glucose 6-phosphate	HMDB0001401	C00092	Organooxygen compounds	MWDB + MS/MS	2	Negative	1.08	2.24	up	CTX vs. H-GLPP
N'-Formylkynurenine	HMDB0001200	C02406	Organooxygen compounds	MWDB + MS/MS	2	Negative	1.07	2.11	up	CTX vs. H-GLPP
3-(3-Hydroxyphenyl)-3-hydroxypropanoic acid	-	-	Phenylpropanoic acids	MWDB + MS/MS	2	Negative	1.16	0.44	down	CTX vs. H-GLPP
2'-Deoxyinosine	HMDB0000071	C05512	Purine nucleosides	MWDB + MS/MS	2	Positive	1.14	0.47	down	CTX vs. H-GLPP
Deoxyadenosine	HMDB0000101	C00559	Purine nucleosides	MWDB + MS/MS	2	Positive	1.13	0.5	down	CTX vs. H-GLPP
Corticosterone	HMDB0001547	C02140	Steroids and steroid derivatives	MWDB + MS/MS	2	Negative	1.15	0.48	down	CTX vs. H-GLPP
Hippuric acid	HMDB0000714	C01586	Benzene and substituted derivatives	MWDB + MS/MS	2	Positive	1.1	6.84	up	CTX vs. H-GLPP
Indole-3-pyruvic acid	HMDB0060484	C00331	-	MWDB + MS/MS	2	Negative	1.11	2.14	up	CTX vs. LMS
Nicotinic acid adenine dinucleotide	-	C00003	(5'->5')-dinucleotides	MWDB + MS/MS	2	Positive	1.08	2.55	up	CTX vs. LMS
5'-Methylthioadenosine	HMDB0001173	C00170	5'-deoxyribonucleosides	MWDB + MS/MS	2	Positive	1.09	4.24	up	CTX vs. LMS
S-Adenosylmethionine	HMDB0001185	-	5'-deoxyribonucleosides	MWDB + MS/MS	2	Positive	1.09	2.9	up	CTX vs. LMS
2,5-Dihydroxybenzoic acid	HMDB0000152	C00628	Benzene and substituted derivatives	MWDB + MS/MS	2	Negative	1.02	3.33	up	CTX vs. LMS
2,4-Dihydroxybenzoic acid	HMDB0029666	-	Benzene and substituted derivatives	MWDB + MS/MS	2	Negative	1	1234.03	up	CTX vs. LMS
Protocatechuic acid	HMDB0001856	C00230	Benzene and substituted derivatives	MWDB + MS/MS	2	Negative	1.03	24.38	up	CTX vs. LMS
4-Acetamidobutyric acid	HMDB0003681	C02946	Carboxylic acids and derivatives	MWDB + MS/MS	2	Positive	1.13	2.47	up	CTX vs. LMS
Creatine	HMDB0000064	C00300	Carboxylic acids and derivatives	MWDB + MS/MS	2	Negative	1.12	0.27	down	CTX vs. LMS
N-Acetylalanine	HMDB0000766	-	Carboxylic acids and derivatives	MWDB + MS/MS	2	Positive	1.11	0.27	down	CTX vs. LMS
p-Coumaraldehyde	HMDB0040986	C05608	Cinnamaldehydes	MWDB + MS/MS	2	Positive	1.01	3.45	up	CTX vs. LMS
Marmesin	HMDB0030786	C09276	Coumarins and derivatives	MWDB + MS/MS	2	Negative	1.09	3.88	up	CTX vs. LMS
5-HEPE	-	-	Fatty Acyls	MWDB + MS/MS	2	Negative	1.05	2.42	up	CTX vs. LMS
Dodecanedioic acid	HMDB0000623	C02678	Fatty Acyls	MWDB + MS/MS	2	Negative	1.14	2.38	up	CTX vs. LMS
9-HpODE	HMDB0006940	C14827	Fatty Acyls	MWDB + MS/MS	2	Negative	1.05	2.97	up	CTX vs. LMS
Palmitoylcarnitine	HMDB0000222	C02990	Fatty Acyls	MWDB + MS/MS	2	Positive	1.13	0.04	down	CTX vs. LMS
Acetylcarnitine	HMDB0000201	C02571	Fatty Acyls	MWDB + MS/MS	2	Positive	1.07	0.29	down	CTX vs. LMS
Leukotriene D4	HMDB0003080	C05951	Fatty Acyls	MWDB + MS/MS	2	Positive	1.11	0.1	down	CTX vs. LMS
Flavin adenine dinucleotide	HMDB0001248	C00016	Flavin nucleotides	MWDB + MS/MS	2	Negative	1.07	2.09	up	CTX vs. LMS
LysoPA 16:0	-	C00416	Glycerophospholipids	MWDB + MS/MS	2	Negative	1.33	0.46	down	CTX vs. LMS
LysoPE 18:0	-	-	Glycerophospholipids	MWDB + MS/MS	2	Negative	1.04	0.38	down	CTX vs. LMS
LysoPC 20:2	-	-	Glycerophospholipids	MWDB + MS/MS	2	Positive	1.35	0.38	down	CTX vs. LMS
LysoPC 14:0	-	-	Glycerophospholipids	MWDB + MS/MS	2	Positive	1.33	0.46	down	CTX vs. LMS
LysoPC 18:1	-	-	Glycerophospholipids	MWDB + MS/MS	2	Positive	1.31	0.49	down	CTX vs. LMS
LysoPC 20:1	-	-	Glycerophospholipids	MWDB + MS/MS	2	Positive	1.31	0.37	down	CTX vs. LMS
LysoPC 16:0	-	-	Glycerophospholipids	MWDB + MS/MS	2	Positive	1.3	0.45	down	CTX vs. LMS
LysoPC 16:1	-	-	Glycerophospholipids	MWDB + MS/MS	2	Positive	1.3	0.32	down	CTX vs. LMS
LysoPC 18:3	-	-	Glycerophospholipids	MWDB + MS/MS	2	Positive	1.28	0.44	down	CTX vs. LMS
LysoPC 18:0	-	-	Glycerophospholipids	MWDB + MS/MS	2	Positive	1.27	0.31	down	CTX vs. LMS
PAF C-16	-	-	Glycerophospholipids	MWDB + MS/MS	2	Positive	1.26	0.31	down	CTX vs. LMS
LysoPC 15:0	-	-	Glycerophospholipids	MWDB + MS/MS	2	Positive	1.24	0.36	down	CTX vs. LMS
LysoPC 17:0	-	-	Glycerophospholipids	MWDB + MS/MS	2	Positive	1.24	0.38	down	CTX vs. LMS
Glycerophosphatidylcholine	HMDB0000086	C00670	Glycerophospholipids	MWDB + MS/MS	2	Positive	1.04	0.22	down	CTX vs. LMS
Lactic acid	HMDB0000190	C00186	Hydroxy acids and derivatives	MWDB + MS/MS	2	Negative	1.16	0.5	down	CTX vs. LMS
Theobromine	HMDB0002825	C07480	Imidazopyrimidines	MWDB + MS/MS	2	Positive	1.12	0.49	down	CTX vs. LMS
5-Hydroxyindole-3-acetic acid	HMDB0000763	C05635	Indoles and derivatives	MWDB + MS/MS	2	Positive	1.03	5.67	up	CTX vs. LMS
3-Indolepropionic acid	HMDB0002302	-	Indoles and derivatives	MWDB + MS/MS	2	Negative	1.09	2.19	up	CTX vs. LMS
Tryptophol	HMDB0003447	C00955	Indoles and derivatives	MWDB + MS/MS	2	Negative	1.07	0.31	down	CTX vs. LMS
Erythrono-1,4-lactone	HMDB0000349	-	Lactones	MWDB + MS/MS	2	Negative	1.12	2.38	up	CTX vs. LMS
Spermidine	HMDB0001257	C00315	Organonitrogen compounds	MWDB + MS/MS	2	Positive	1.09	5.34	up	CTX vs. LMS

3-Methyl-1-butylamine	-	C02640	Organonitrogen compounds	MWDB + MS/MS	2	Positive	1.07	2553.39	up	CTX vs. LMS
N-Acetylglucosamine 1-phosphate	HMDB0001367	C04256	Organooxygen compounds	MWDB + MS/MS	2	Negative	1.13	0.45	down	CTX vs. LMS
N-Acetylneuraminic acid	HMDB0000230	C19910	Organooxygen compounds	MWDB + MS/MS	2	Positive	1.17	0.47	down	CTX vs. LMS
3-(3-Hydroxyphenyl)propionic acid	HMDB0000375	C11457	Phenylpropanoic acids	MWDB + MS/MS	2	Negative	1.07	8.95	up	CTX vs. LMS
3-Phenyllactic acid	HMDB0000779	C05607	Phenylpropanoic acids	MWDB + MS/MS	2	Negative	1.06	4.7	up	CTX vs. LMS
Hydrocinnamic acid	HMDB0000764	C05629	Phenylpropanoic acids	MWDB + MS/MS	2	Negative	1	4.62	up	CTX vs. LMS
11-cis-Retinol	HMDB0006216	C00899	Prenol lipids	MWDB + MS/MS	2	Positive	1.03	5.98	up	CTX vs. LMS
ADP-ribose	HMDB0001178	C00301	Purine nucleosides	MWDB + MS/MS	2	Negative	1.1	2.13	up	CTX vs. LMS
N6-Succinyl adenosine	-	-	Purine nucleosides	MWDB + MS/MS	2	Positive	1.25	0.45	down	CTX vs. LMS
Pyridoxine	HMDB0000239	C00314	Pyridines and derivatives	MWDB + MS/MS	2	Positive	1.1	3.51	up	CTX vs. LMS
Nicotinamide	HMDB0001406	C00153	Pyridines and derivatives	MWDB + MS/MS	2	Positive	1.07	2.3	up	CTX vs. LMS
6-Methylnicotinamide	HMDB0013704	-	Pyridines and derivatives	MWDB + MS/MS	2	Positive	1.16	2.13	up	CTX vs. LMS
Pyrrole-2-carboxylic acid	HMDB0004230	C05942	Pyrroles	MWDB + MS/MS	2	Negative	1.05	2.92	up	CTX vs. LMS
Xanthurenic acid	HMDB0000881	C02470	Quinolines and derivatives	MWDB + MS/MS	2	Positive	1.02	9.63	up	CTX vs. LMS
Taurochenodesoxycholic acid	HMDB0000951	C05465	Steroids and steroid derivatives	MWDB + MS/MS	2	Negative	1.02	0.35	down	CTX vs. LMS
Estrone 3-sulfate	HMDB0001425	C02538	Steroids and steroid derivatives	MWDB + MS/MS	2	Negative	1.01	5.23	up	CTX vs. LMS
