# Supplementary figures and images for: Integrated Foodomics Reveals Gut Microbiota–Metabolite–Gene Interactions Associated with the Immunoprotective Effects of Ganoderma lucidum Polysaccharide Peptide
Source: Foods. 2026 Jul 3;15(13):2370. doi: 10.3390/foods15132370 (PMC13361114; doi:10.3390/foods15132370)

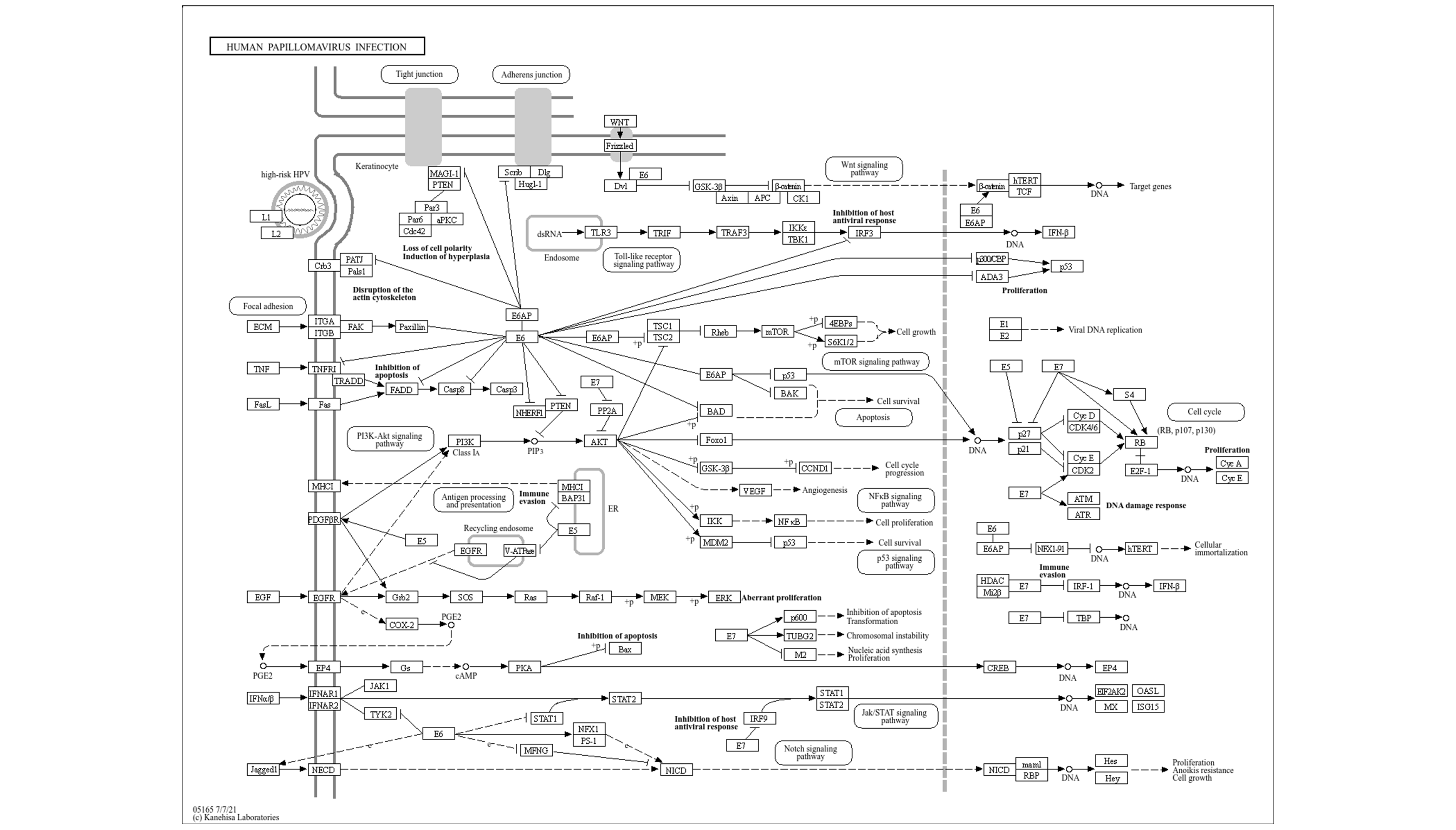

Supplement: Supplementary file 1 [file foods-15-02370-s001.zip › Fig S1.tif]

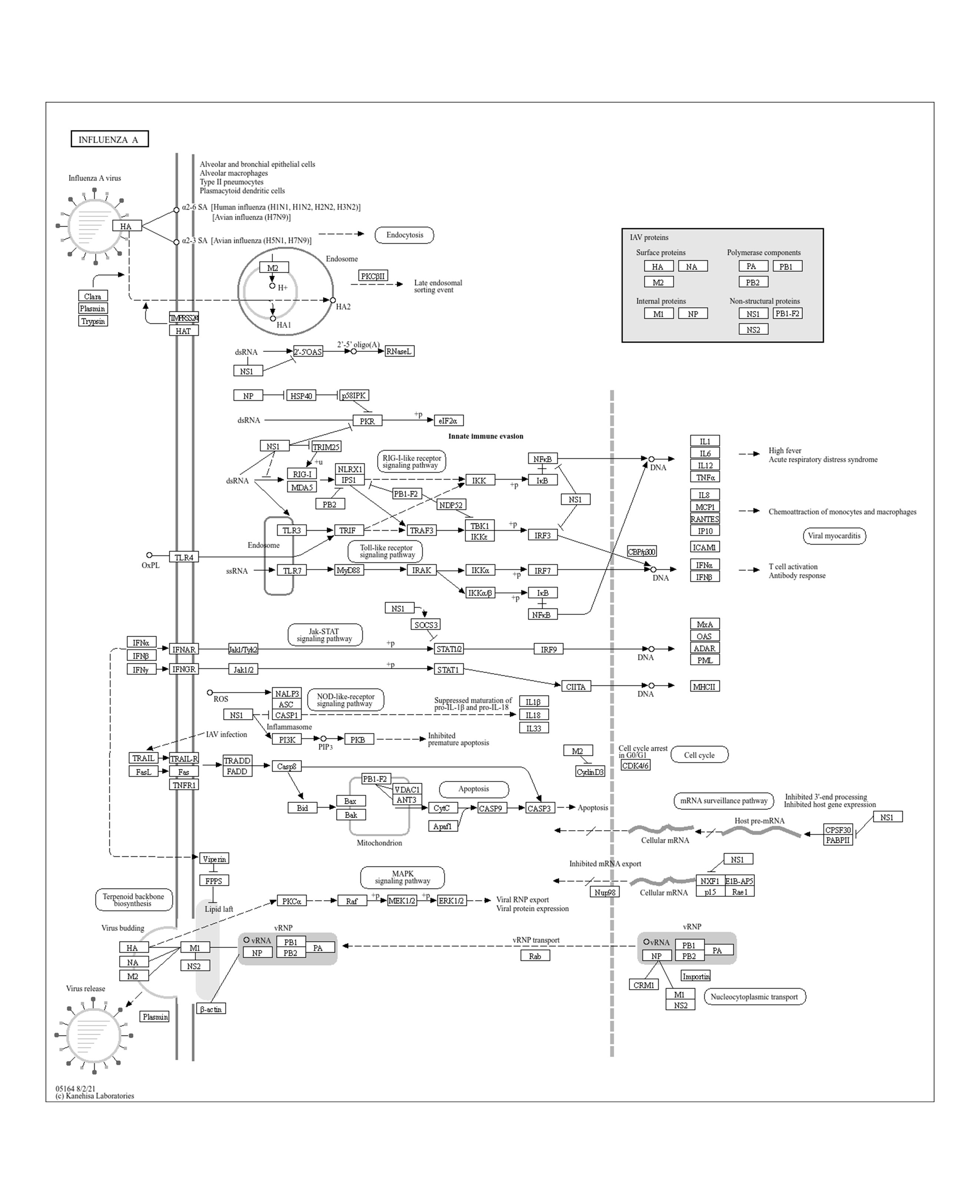

Supplement: Supplementary file 1 [file foods-15-02370-s001.zip › Fig S2.tif]

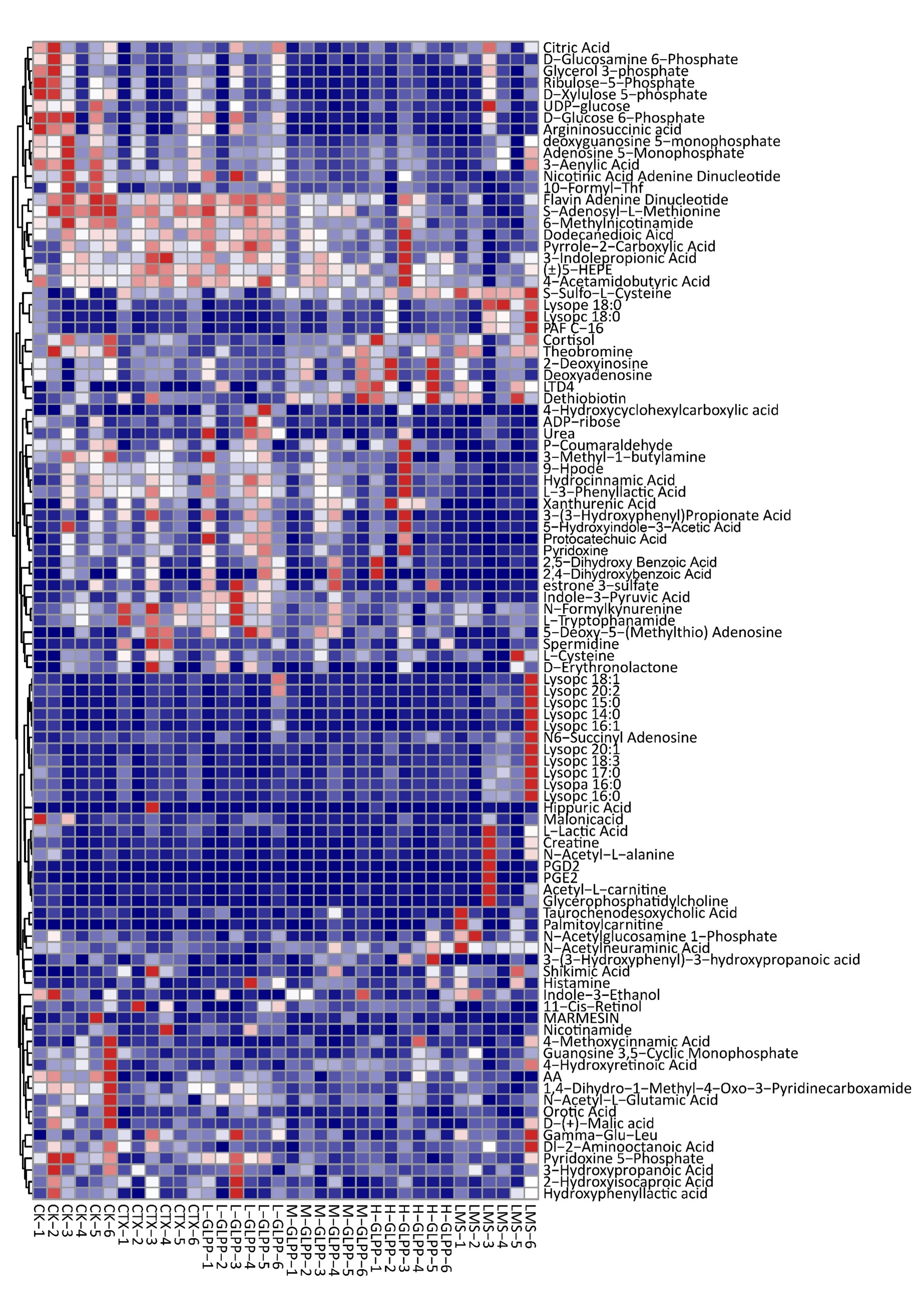

Supplement: Supplementary file 1 [file foods-15-02370-s001.zip › Fig S3A.tif]

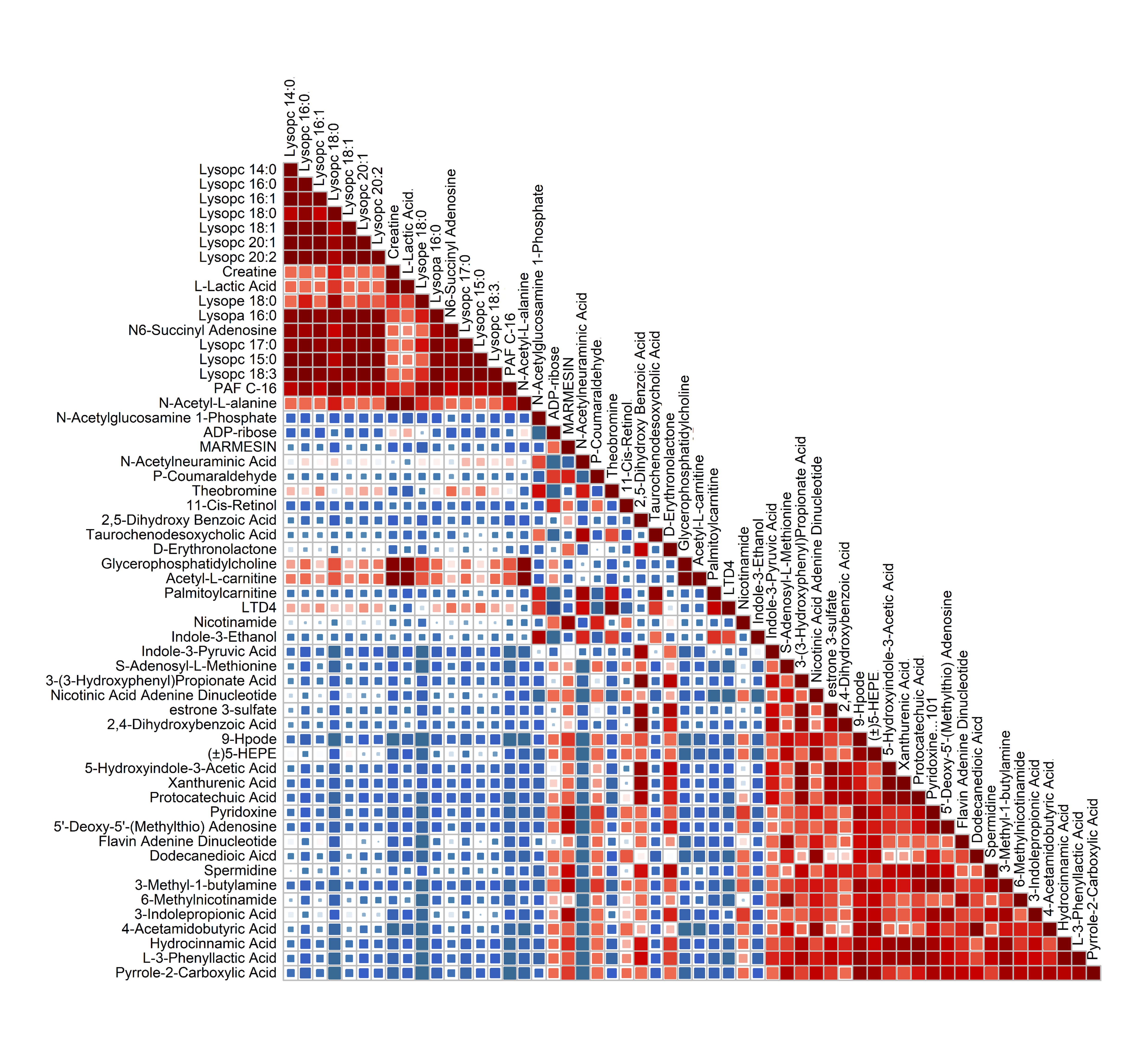

Supplement: Supplementary file 1 [file foods-15-02370-s001.zip › Fig S3B.tif]

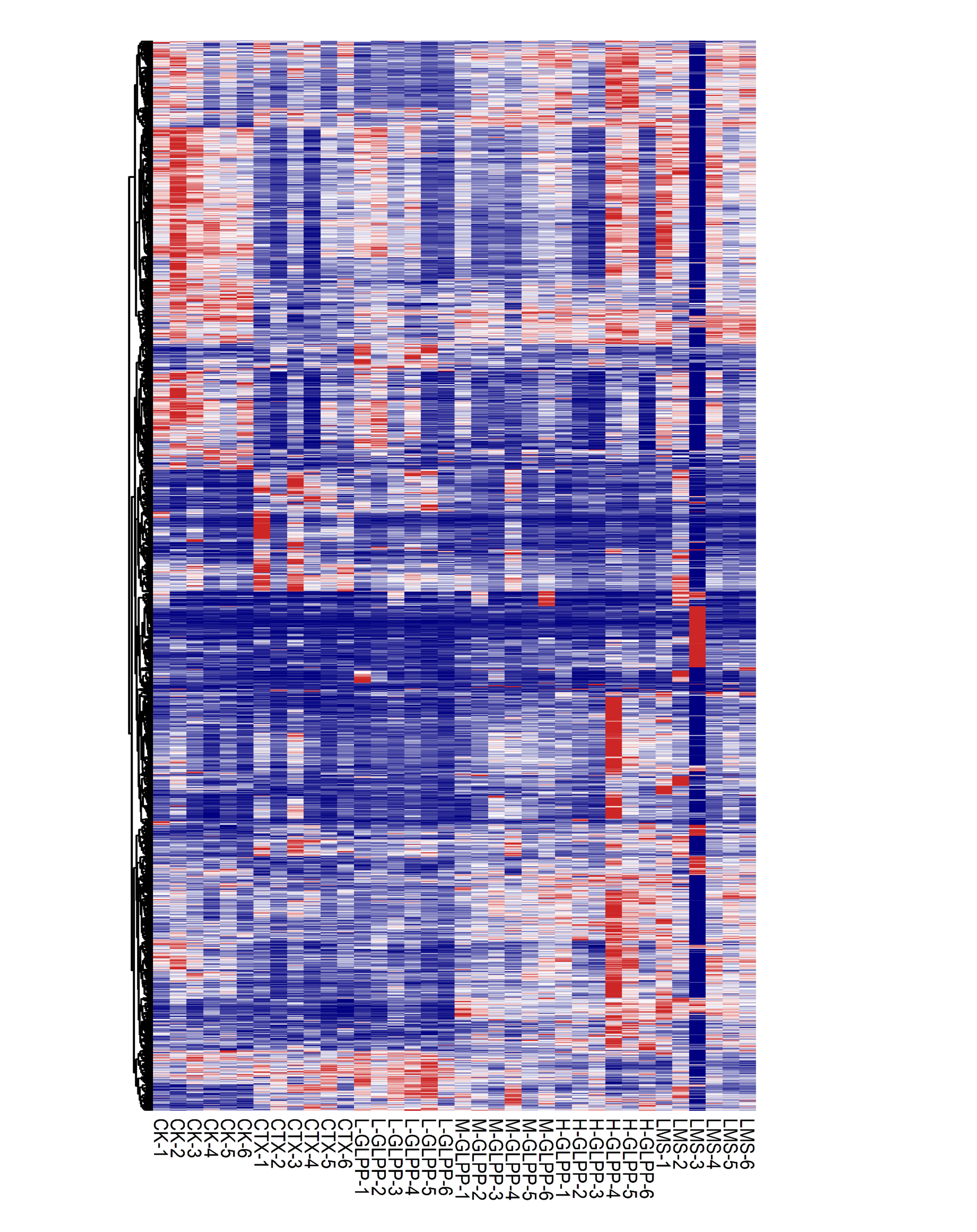

Supplement: Supplementary file 1 [file foods-15-02370-s001.zip › Fig S4.tif]

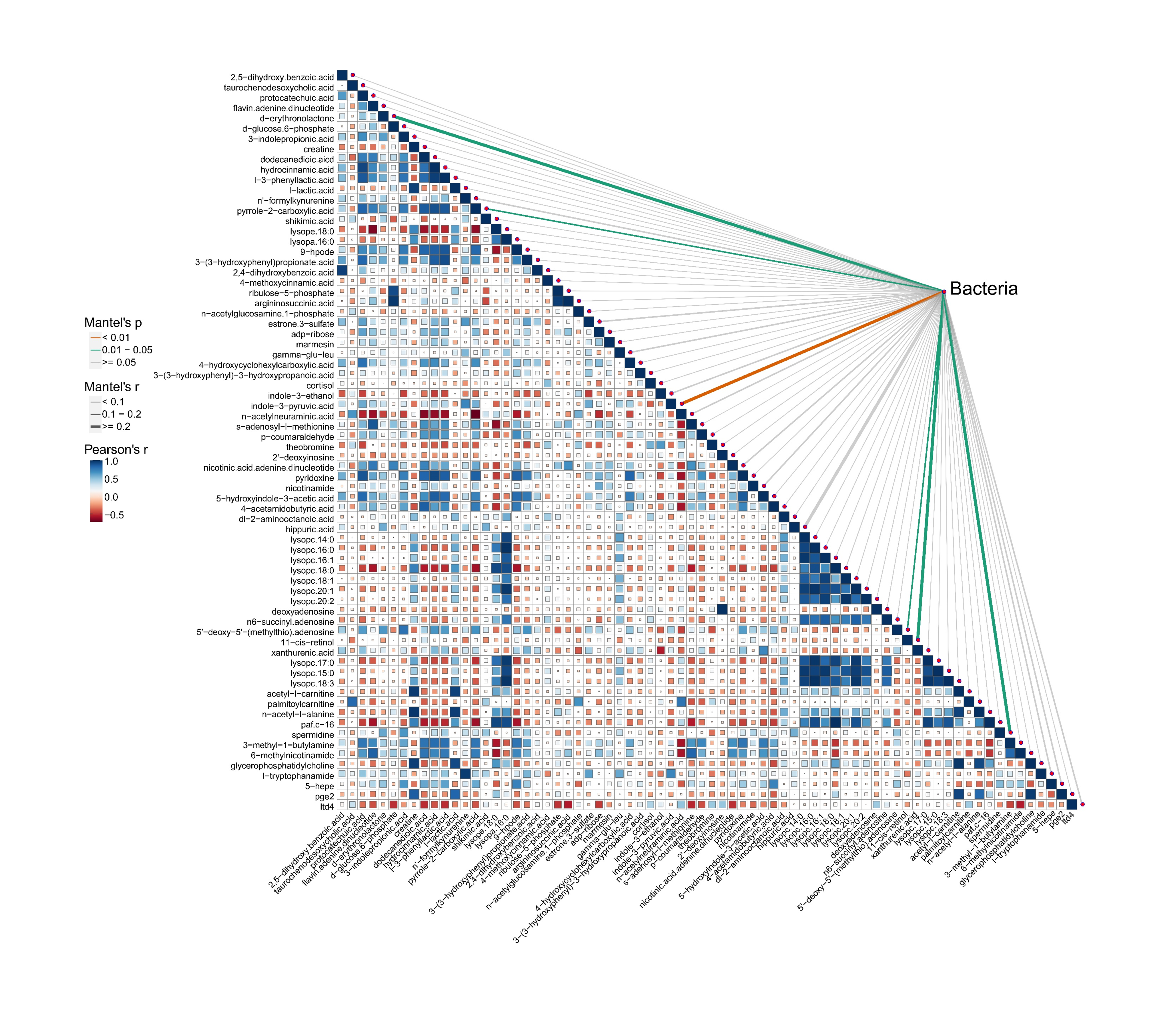

Supplement: Supplementary file 1 [file foods-15-02370-s001.zip › Fig S5A.tif]

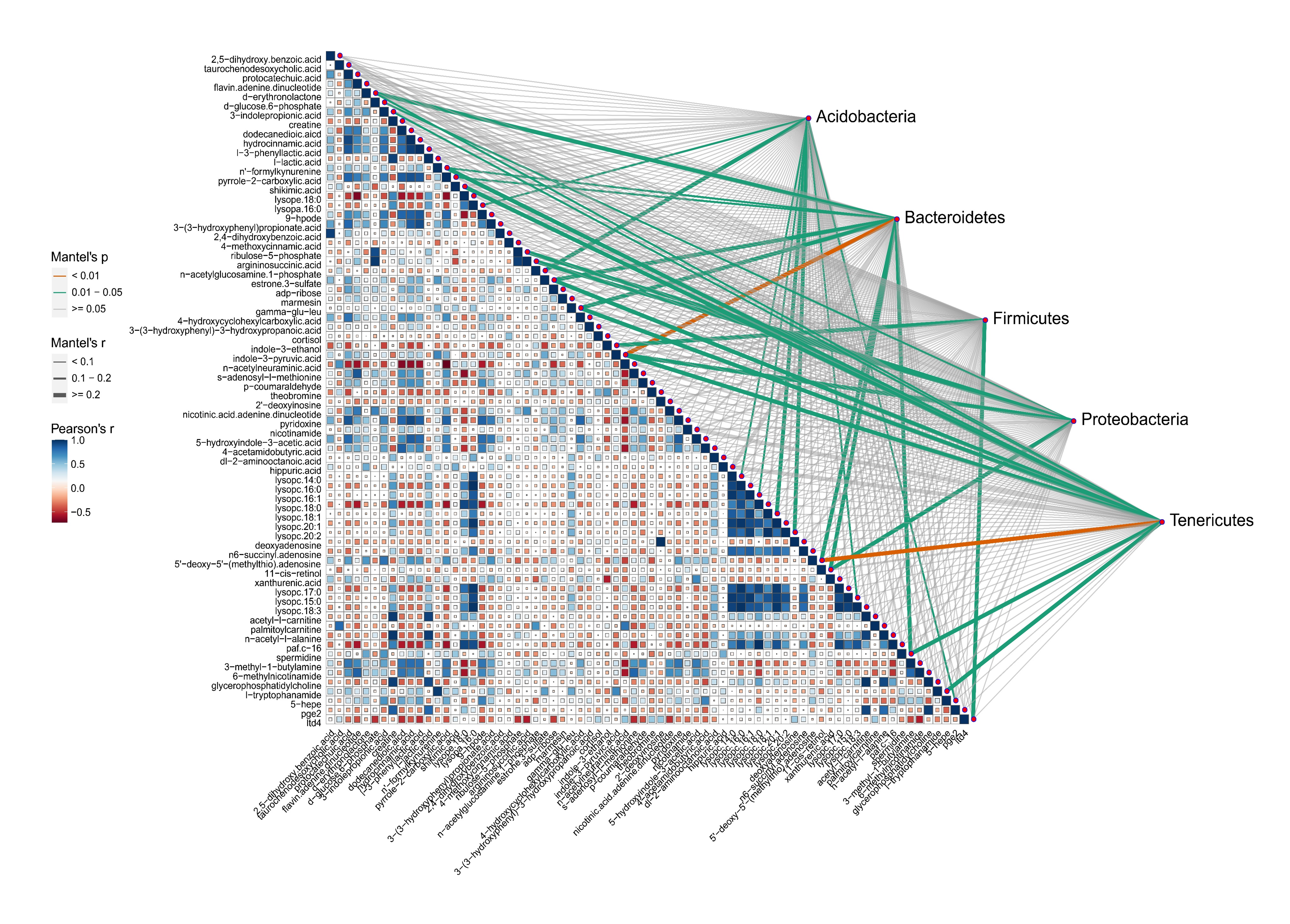

Supplement: Supplementary file 1 [file foods-15-02370-s001.zip › Fig S5B.tif]

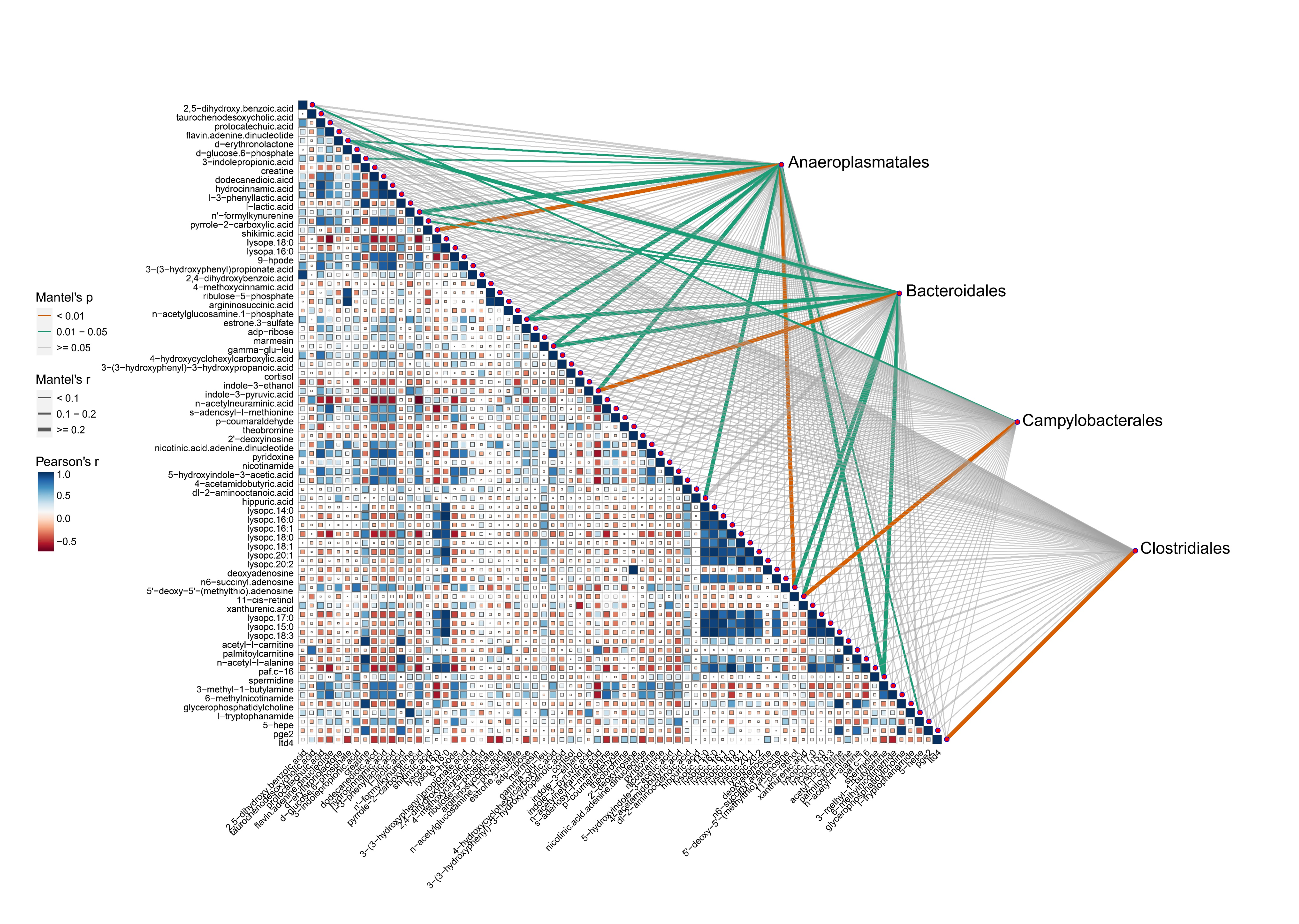

Supplement: Supplementary file 1 [file foods-15-02370-s001.zip › Fig S5C.tif]

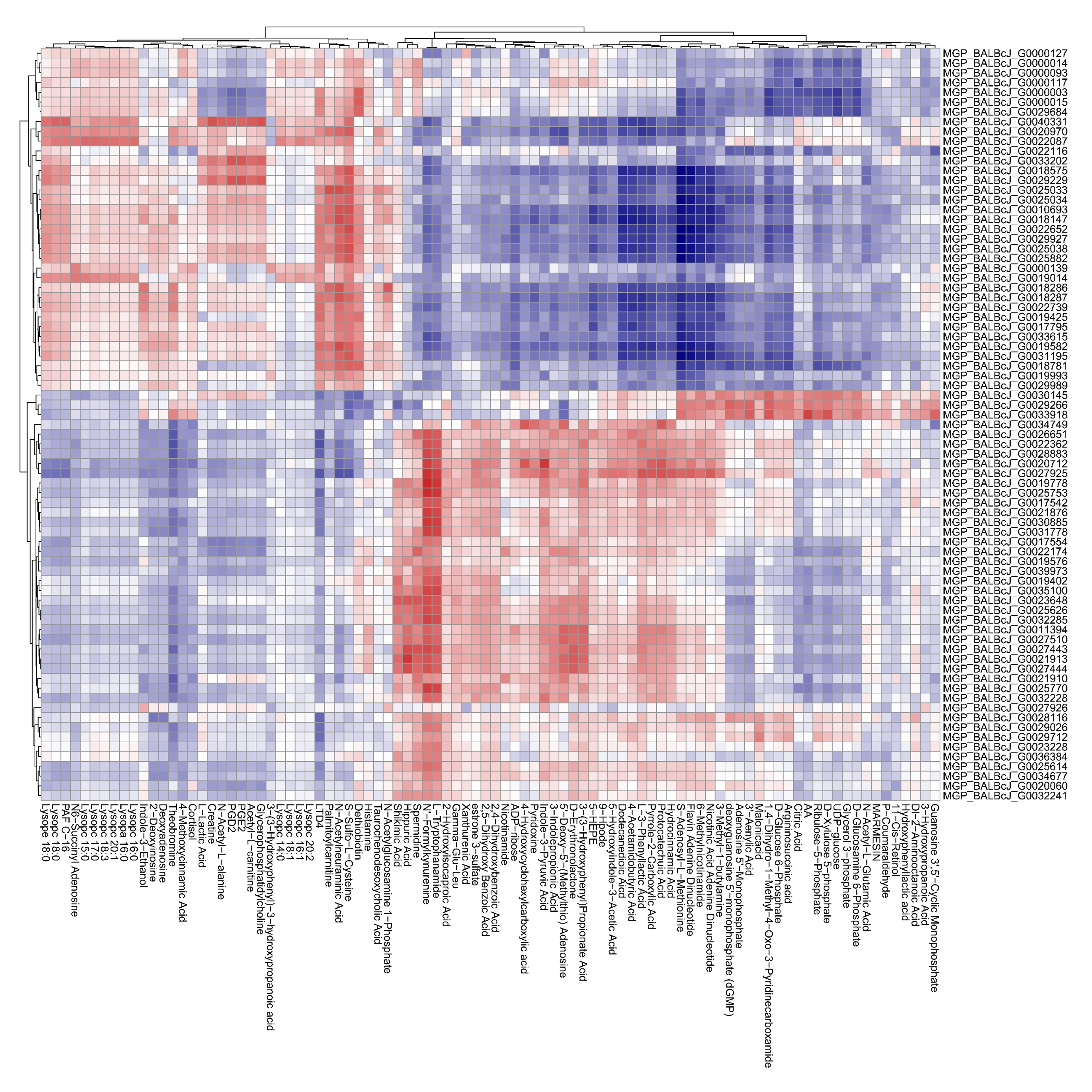

Supplement: Supplementary file 1 [file foods-15-02370-s001.zip › Fig S5D.tif]
